# Immunometabolic alterations in young adults with obesity are accompanied by divergent monocyte and muscle mitochondrial bioenergetic profiles

**DOI:** 10.1007/s44357-026-00031-5

**Published:** 2026-11-14

**Authors:** Spencer Ursel, Sriyashika Nekkanti, Kimberly Day, Roderick E. Sandilands, Dylan Da Silva, Raj Ahluwalia, Ella Harness, Alexandre Abílio de Souza Teixeira, Travis D. Gibbons, Jared Baylis, Bryon R. McKay, Robert Boushel, Jonathan P. Little, Hashim Islam

**Affiliations:** 1 School of Health & Exercise Sciences, University of British Columbia, Kelowna, BC Canada; 2 Immunometabolism Research Group, Institute of Biomedical Sciences, University of São Paulo (USP), São Paulo, Brazil; 3 Department of Biological Sciences, Northern Arizona University, Flagstaff, AZ USA; 4 Department of Emergency Medicine, University of British Columbia, Vancouver, BC Canada; 5 Department of Ophthalmology and Visual Sciences, University of British Columbia, Vancouver, BC Canada; 6 Department of Kinesiology, University of British Columbia, Vancouver, BC Canada; 7 Centre for Chronic Disease Prevention & Management, University of British Columbia, Kelowna, BC Canada

**Keywords:** Glycaemic management, Immunometabolism, Inflammation, Insulin sensitivity, Mitochondria, Obesity

## Abstract

**Aims/hypothesis:**

Mitochondrial dysfunction and chronic inflammation are implicated in obesity-associated metabolic disorders. Studies interrogating muscle mitochondrial bioenergetics in obesity are confounded by the impact of age and/or physical activity. How obesity impacts mitochondrial bioenergetics in monocytes—precursors to muscle-resident macrophages—is poorly understood. We aimed to compare whole-body immunometabolic function and mitochondrial respiration in muscle and monocytes in adults with or without obesity of similar sex, age and physical activity levels.

**Methods:**

In this cross-sectional study, young adults with obesity (BMI ≥ 30) or without obesity (BMI < 30) of similar age, sex and activity levels provided a fasted blood sample and muscle biopsy (vastus lateralis), and underwent a mixed-meal tolerance test. Mitochondrial respiration was measured in permeabilised muscle fibres and blood monocytes using high-resolution respirometry alongside muscle mitochondrial proteins and enzyme activity. Blood monocyte phenotype, polarisation and mitochondrial reactive oxygen species (mtROS) were quantified using flow cytometry.

**Results:**

Young adults with obesity (*n* = 17 [9 female/8 male], age 23.1 [SD 5.7] years, BMI 33.2 [SD 3.1] kg/m^2^) exhibited impaired whole-body insulin sensitivity (*d* = 1.2) and elevated white blood cell counts (*d* = 0.9; both *p* < 0.01) compared with those without obesity (*n* = 20 [10 female/10 male], age 22.8 [SD 4.6] years, BMI 21.8 [SD 2.1] kg/m^2^). Muscle oxidative phosphorylation (OXPHOS; *d* = 0.7–0.8) and electron transfer system capacity (ETS; *d* = 1.0) were higher in young adults with obesity than in those without obesity (*p* < 0.05) despite similar mitochondrial content markers (*d* = 0.2; p > 0.6). Monocyte OXPHOS (*d* = 1.0) and ETS (*d* = 0.8) were attenuated in those with obesity and accompanied by lower Toll-like receptor 4 expression (*d* = 0.7–0.8), diminished cytokine-stimulated mtROS production (*d* = 0.8–1.0) (all *p* < 0.05) and exaggerated proinflammatory marker expression ex vivo (*d* = 0.8; p = 0.07).

**Conclusions/interpretation:**

Young adults with obesity exhibit elevated muscle mitochondrial respiration—consistent with early adaptive remodelling in response to energy surplus—accompanied by heightened systemic inflammation and cellular markers suggestive of a dysregulated immunometabolic phenotype.

**Graphical Abstract:**

**Figure:**
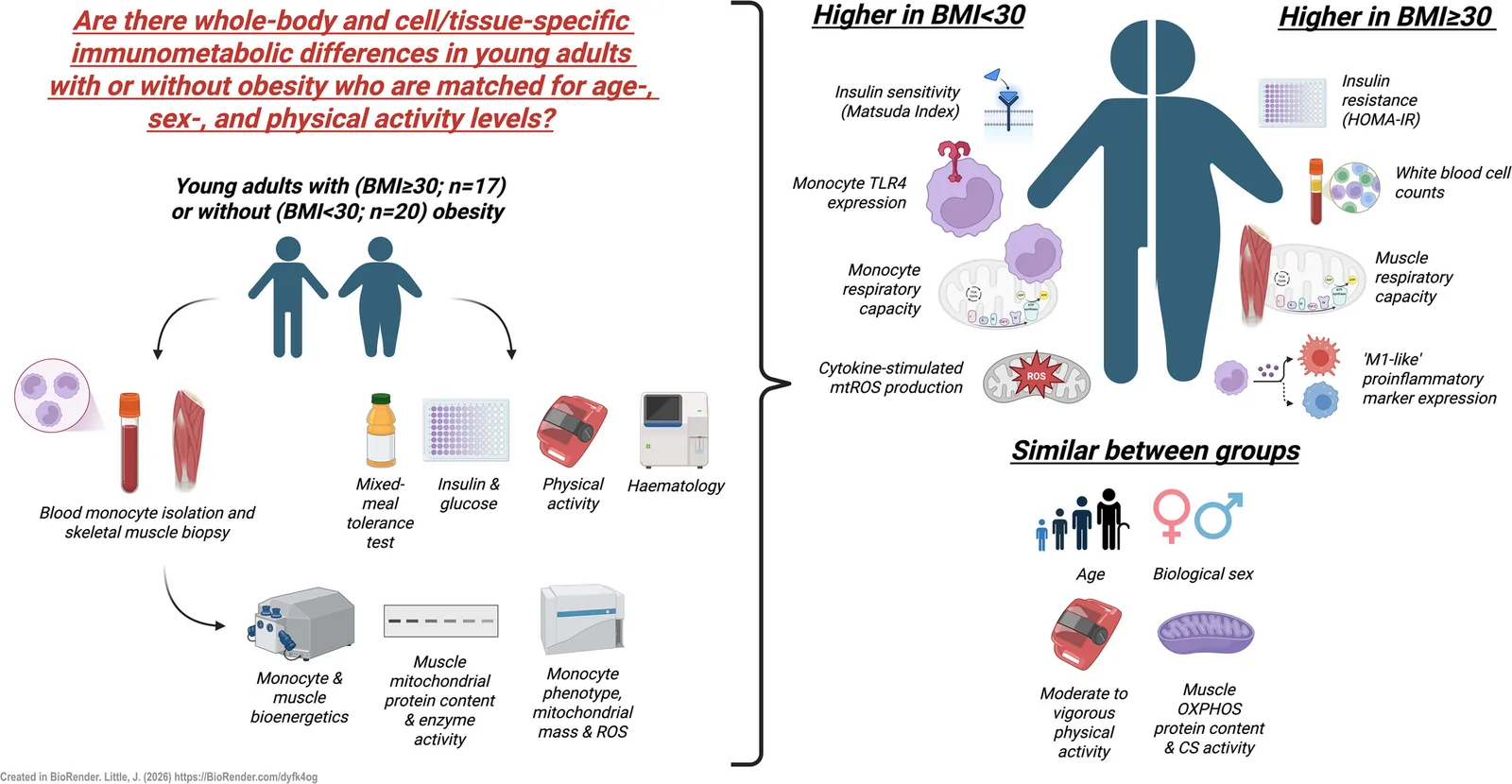

**Supplementary Information:**

The online version contains supplementary material available at https://doi.org/10.1007/s44357-026-00031-5.

**Figure:**
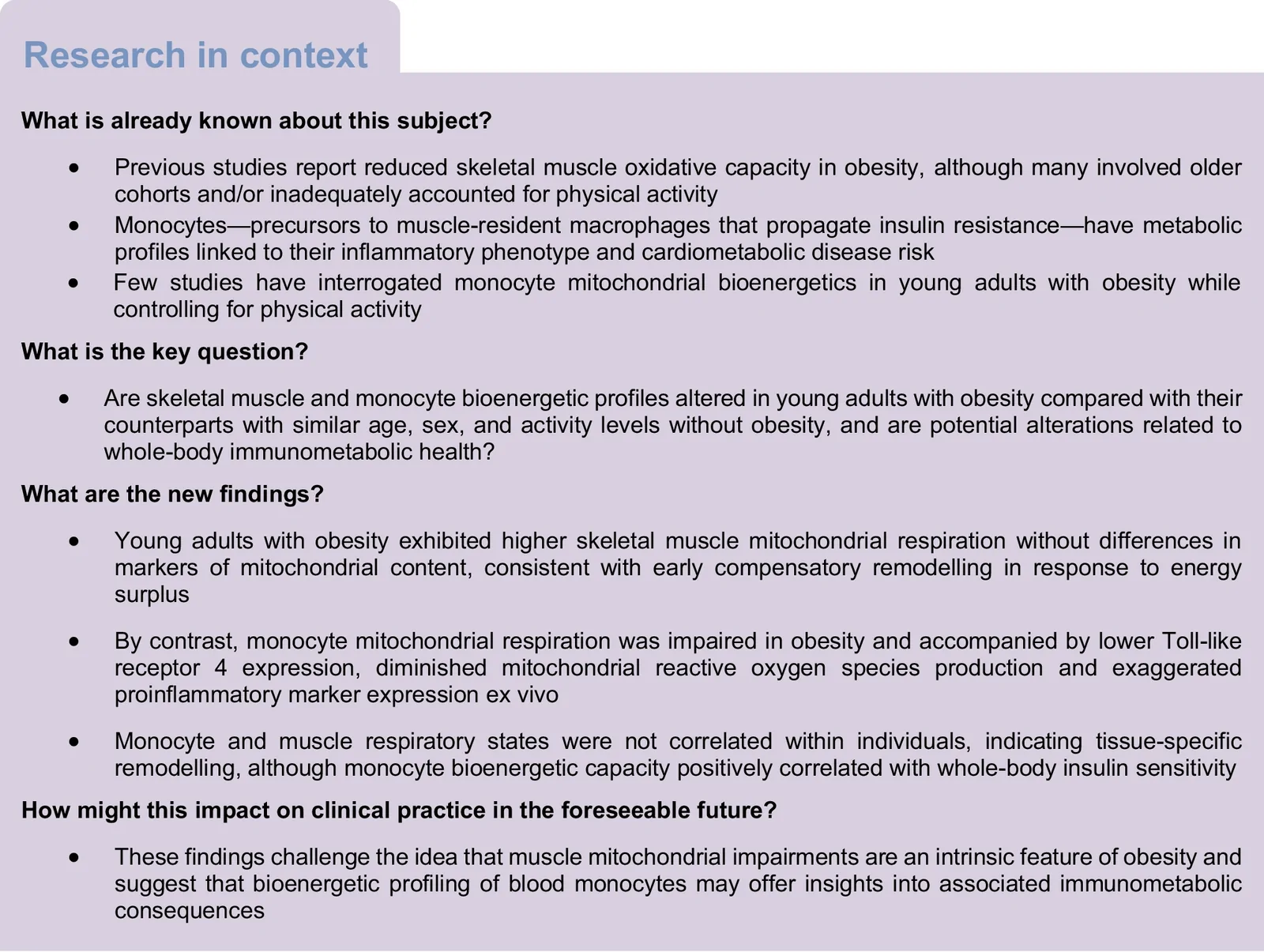

## Introduction

Prior studies report mitochondrial defects characterised by reduced oxidative capacity and/or altered substrate utilisation in skeletal muscle from individuals with obesity [1–7]. These deficits have been linked to impaired insulin signalling, potentially contributing to the development of insulin resistance and type 2 diabetes [6, 8–10]. Animal studies indicate that high-fat diets can induce mitochondrial biogenesis—a finding that challenges the causative role of obesity-induced mitochondrial deficits in the pathogenesis of type 2 diabetes [11, 12]. These findings warrant further investigation into how obesity impacts muscle mitochondrial metabolism in relation to whole-body insulin sensitivity in humans.

Most studies interrogating the impact of obesity on muscle mitochondrial bioenergetics have involved older populations (e.g. those with more severe insulin resistance or type 2 diabetes) [4, 6, 9, 13–15], which limits insights into early mitochondrial alterations associated with obesity. In light of rodent data showing early compensatory increases in mitochondrial capacity in response to energy surplus [11, 16] and the reported mitochondrial deficits in aged muscle [17, 18], studying younger cohorts with obesity may help to unravel early changes in muscle mitochondrial capacity that precede subsequent bioenergetic decline. Furthermore, existing studies examining muscle mitochondrial profiles in obesity do not appear to adequately account for physical activity levels [1, 4, 9, 15, 19], as indicated by group-level differences in self-reported activity and measured fitness and/or training history and lack objective accelerometry-based activity assessments. This is an important limitation as physical activity and exercise are powerful stimuli for skeletal muscle mitochondrial adaptation [20, 21]. Several of these studies have also included older cohorts [2–4, 14, 15], which could also contribute to reported mitochondrial deficits [17, 18]. Interrogating mitochondrial bioenergetics in young adults with obesity, while controlling for physical activity, could provide insights into the associated impact of obesity per se.

Chronic low-grade inflammation is another related feature of obesity that is hypothesised to propagate insulin resistance [22, 23]. Monocytes are key innate cells involved in the inflammatory response, and are precursors to resident macrophages that propagate insulin resistance in key metabolic tissues such as skeletal muscle [24, 25]. Recent work has also highlighted a broader role for muscle-resident immune cells, including macrophages [26], in skeletal muscle metabolic homeostasis, regulation of mitochondrial integrity and exercise-induced metabolic adaptation through intercellular crosstalk within the muscle microenvironment [27]. Monocyte metabolism is linked to their immunophenotype [28, 29], with proinflammatory cells, such as those that infiltrate muscle and propagate insulin resistance, generally exhibiting reduced mitochondrial oxidative phosphorylation (OXPHOS) capacity [30]. Importantly, monocyte mitochondrial profiles are coupled to whole-body insulin resistance in mice [31, 32] and predict cardiometabolic disease risk in humans with type 2 diabetes [31]. Few studies have interrogated how obesity impacts monocyte mitochondrial bioenergetics [29, 33] and these studies have again involved older cohorts, which may confound interpretation of the impacts of obesity per se (similar to muscle). Importantly, studying mitochondrial bioenergetics in both circulating monocytes—the precursors to muscle-resident macrophages—and skeletal muscle within the same individuals provides a way to better understand the immunometabolic impacts of obesity across two key tissues with established bidirectional crosstalk.

To address the aforementioned limitations and expand understanding of how obesity impacts mitochondrial bioenergetics, the purpose of this study was to assess skeletal muscle and monocyte mitochondrial respiration in young adults with obesity compared their counterparts without obesity of similar age, sex and physical activity levels. To link muscle and monocyte mitochondrial respiration to whole-body metabolic health, we also conducted mixed-meal tolerance tests to determine insulin sensitivity, and determined relationships using correlation analyses. To explore how metabolic capacity may impact monocyte inflammatory profiles and immunophenotypes in obesity, we classified major blood monocyte subsets and cultured isolated monocytes to determine reactive oxygen species (ROS) production and phenotype shifts in response to ex vivo cytokine stimulation. Our work provides new insights into the immunometabolic consequences of obesity, which could help to advance understanding of obesity pathophysiology and its associated complications.

## Methods

### Study design, participants and laboratory visits

This cross-sectional study enrolled healthy adults with obesity (BMI ≥ 30) and without obesity (BMI < 30) from Kelowna, BC, Canada. Groups were similar in age, sex, and physical activity assessed by waist-worn accelerometry (ActiGraph GT3X). Inclusion criteria were as follows: (1) 19–40 years of age; (2) BMI 18.5–40 kg/m^2^; (3) free of cardiometabolic (e.g. heart disease, diabetes) and autoimmune/inflammatory (e.g. colitis) disease; and (4) ability to fast overnight. Participants were excluded if they: (1) were cigarette smokers; (2) were taking immunomodulatory/anti-inflammatory medications; (3) self-reported engagement in more than 150 min of weekly physical activity and/or were highly trained endurance athletes based on self-reported data; (4) were currently pregnant; (5) were unable to travel to/from the university; or (6) were unable to read or communicate in English. All participants provided written informed consent in accordance with the Declaration of Helsinki. The study was approved by The University of British Columbia Clinical Research Ethics Board (H22-03654) and pre-registered in Open Science Framework (https://doi.org/10.17605/OSF.IO/JPSKU).

Eligible participants abstained from alcohol and structured exercise for 24 h and arrived after an overnight fast (≥ 8 h) for two separate laboratory visits conducted at the Centre for Chronic Disease Prevention and Management at the University of British Columbia (Okanagan). One of the visits involved the completion of questionnaires, body composition assessments and a fasted muscle biopsy, whereas the other visit involved a mixed-meal tolerance test (details below). Height was measured via stadiometer. Total body mass, lean mass and fat mass were assessed using bioimpedance analysis (InBody 770). Participants were also provided with a waist-worn accelerometer for a 7-day period to objectively measure physical activity levels (details below). Analyses of biological samples were performed with samples labelled only by participant ID to blind outcome assessors to participant BMI category.

### Outcomes and analyses

Experimental details for each outcome are provided in electronic supplementary material (ESM) Methods. During one of the experimental visits, a fasted muscle biopsy (vastus lateralis) was obtained using the modified Bergström technique, with a portion of muscle used fresh for high-resolution respirometry and the remainder snap frozen for analyses of mitochondrial protein content and enzyme activity analyses. On a separate visit, an indwelling i.v. catheter was used to collect a fasted blood sample, followed by a mixed-meal tolerance test with additional sampling at 15, 30, 60, 90 and 120 min postprandially. Baseline (fasted) blood was used to isolate CD14 + monocytes via immunomagnetic negative selection, determine blood monocyte subsets, phenotype and ROS production and to determine complete blood counts. Plasma was isolated from all blood samples to determine insulin and glucose concentrations. Physical activity was measured using 7-day waist-worn accelerometry (full processing parameters are detailed in the ESM Methods).

Mitochondrial respiration was assessed in permeabilised muscle fibres and isolated CD14 + monocytes by high-resolution respirometry using established substrate-uncoupler-inhibitor titration protocols. Substrate/inhibitor concentrations and titration steps for both tissues are detailed in ESM Table 3 and ESM Table 4. Citrate synthase maximal activity, a validated marker of mitochondrial content, was measured spectrophotometrically in whole muscle homogenates. Protein content of electron transport chain complex subunits was determined by western blot using a commercial OXPHOS antibody cocktail, with Ponceau red staining used as a loading control. Blood monocyte subsets (classical, intermediate and non-classical) and surface Toll-like receptor 4 (TLR4) expression were determined by flow cytometry based on relative CD14 and CD16 expression. In separate assays, isolated CD14 + monocytes were polarised ex vivo towards proinflammatory (lipopolysaccharide [LPS] + IFNɣ) or anti-inflammatory (IL-10) phenotypes, with CD80 and CD163 surface expression, and mitochondrial mass (MitoTracker Green) and ROS production (MitoSOX Red) assessed in parallel. Antibody panels, catalogue numbers, staining protocols and gating strategies are described in the ESM Methods. Plasma insulin and glucose were measured in duplicate by ELISA and colorimetric assay, respectively, and used to calculate fasting insulin resistance (HOMA-IR) and whole-body insulin sensitivity (Matsuda index) from fasting and postprandial mixed-meal tolerance test values.

### Statistical analysis

Data were visualised using Graph Pad Prism Version 10 and analysed using R (version 4.3.2). Data were assessed for normality and skewness using Shapiro–Wilk test, and Q-Q plots of model residuals were inspected to confirm normality of residuals for statistical analyses. Outcomes with non-normal distributions were log transformed for analyses and back transformed for presentation in figures and tables. Outliers were identified using the robust regression and outlier removal method with a Q value of 1% to minimise the likelihood of excluding valid data points. Differences between groups were determined using linear models adjusted for sex with group as a fixed effect and Cohen’s *d* effect size were calculated (small = 0.3, medium = 0.5, large = 0.8). Pearson’s correlation coefficients were used to determine relationships between select biologically relevant pairings (between monocyte and muscle respiration, and between respiratory parameters and whole-body immune and metabolic markers). Sample size was determined based on previous studies comparing muscle mitochondrial bioenergetics between individuals with obesity and their lean counterparts [1, 15, 19]. Based on a large effect size difference in muscle mitochondrial respiration (primary outcome) in these studies (Cohen’s *d* = 1.0), a sample size of 17 per group was deemed adequate to detect between-group differences with 80% power at an alpha level of 0.05. Significance was accepted as *p* < 0.05.

## Results

### Participant characteristics

Participant characteristics are reported in Table 1 and the sample size for outcomes in each figure (e.g. after outlier removal, sample availability, experiment quality) are included in ESM Table 1. Adjusted means with 95% CIs for each outcome variable are presented in ESM Table 2. As intended, participants with obesity had higher BMI (*d* = 4.4), body fat percentage (*d* = 2.6), visceral fat area (*d* = 4.0) and waist circumference (*d* = 4.2) than those without obesity (all *p* < 0.001). Participants with obesity also exhibited hyperinsulinaemia, with higher plasma insulin concentrations in the fasted state (*p* = 0.004, *d* = 1.1), greater insulin resistance (*p* = 0.006, *d* = 0.9) and lower insulin sensitivity (*p* = 0.003, *d* = 1.2) than those without obesity. Those with obesity also had elevated total white blood cells (*p* = 0.011, *d* = 0.9), lymphocytes (*p* = 0.032, *d* = 0.7) and neutrophils (*p* = 0.046, *d* = 0.7) compared with those without obesity, indicative of a chronic inflammatory state. Both groups were engaging in similar amounts of light (*d* = 0.1), moderate (*d* = 0.2) and vigorous (*d* = 0.1) physical activity per day (*p* > 0.484). Similarly, there were no between-group differences in total daily moderate-to-vigorous physical activity (MVPA) (*p* = 0.940, *d* = 0.2), confirming that the groups were engaging in similar levels of habitual physical activity levels. Notably, the observed age distribution was narrow and well-matched between groups.

**Table 1:** Participant characteristics

Characteristic	Without obesity (BMI < 30; *n* = 20)	With obesity (BMI ≥ 30; *n* = 17)
Age (years)	22.8 ± 4.6	23.1 ± 5.7
Sex (female/male)	10/10	9/8
Body mass (kg)	64.5 ± 10.0	102.2 ± 14.5*
BMI (kg/m^2^)	21.8 ± 2.1	33.2 ± 3.1*
Body fat percentage (%)	19.5 ± 8.2	37.7 ± 4.9*
Visceral fat area (cm^3^)	51.5 ± 29.6	181.8 ± 35.1*
Waist circumference (inches)	29.1 ± 2.4	42.1 ± 3.7*
Systolic BP (mmHg)	118.1 ± 9.6	123.1 ± 13.5
Diastolic BP (mmHg)	69.1 ± 9.0	73.2 ± 9.5
Fasting insulin (µIU/ml)	5.5 ± 3.4	9.5 ± 4.2*
Fasting glucose (mmol/l)	5.42 ± 0.43	5.30 ± 0.52
Inactivity (min)	955 ± 162	933 ± 179
Daily light physical activity (min)	76 ± 46	82 ± 50
Daily moderate physical activity (min)	44 ± 29	50 ± 22
Daily vigorous physical activity (min)	1 ± 2	0.9 ± 1.6
Daily MVPA (bouts ≥ 10 min)	16 ± 14	13 ± 8
HOMA-IR	1.3 ± 0.9	2.3 ± 1.1*
Matsuda Index	11.18 ± 4.6	6.58 ± 2.4*
Total white blood cells (× 10^3^/µl)	5.34 ± 1.13	6.41 ± 1.30*
Lymphocytes (× 10^3^/µl)	1.74 ± 0.36	2.08 ± 0.57*
Monocytes (× 10^3^/µl)	0.43 ± 0.11	0.46 ± 0.13
Neutrophils (× 10^3^/µl)	3.01 ± 1.06	3.70 ± 0.98*
Eosinophils (× 10^3^/µl)	0.14 ± 0.11	0.16 ± 0.06
Basophils (× 10^3^/µl)	0.02 ± 0.01	0.02 ± 0.01
Red blood cells (× 10^6^/µl)	4.71 ± 0.34	4.77 ± 0.59
Haemoglobin (g/dl)	14.62 ± 1.80	14.40 ± 1.35
Haematocrit (%)	42.22 ± 3.49	42.27 ± 4.03
Platelets (× 10^3^/µl)	246.66 ± 60.87	264.79 ± 48.49

Data are means ± SD

^*^p < 0.05 vs BMI < 30 (linear model adjusted for sex; see text and ESM Table 2 for exact *p* values and effect sizes; model adjusted means are provided in ESM Table 2). Glucose and insulin curves from the mixed-meal tolerance test are shown in ESM Figure 1

### Mitochondrial respiration in permeabilised muscle fibres and isolated blood monocytes

**Fig. 1:**
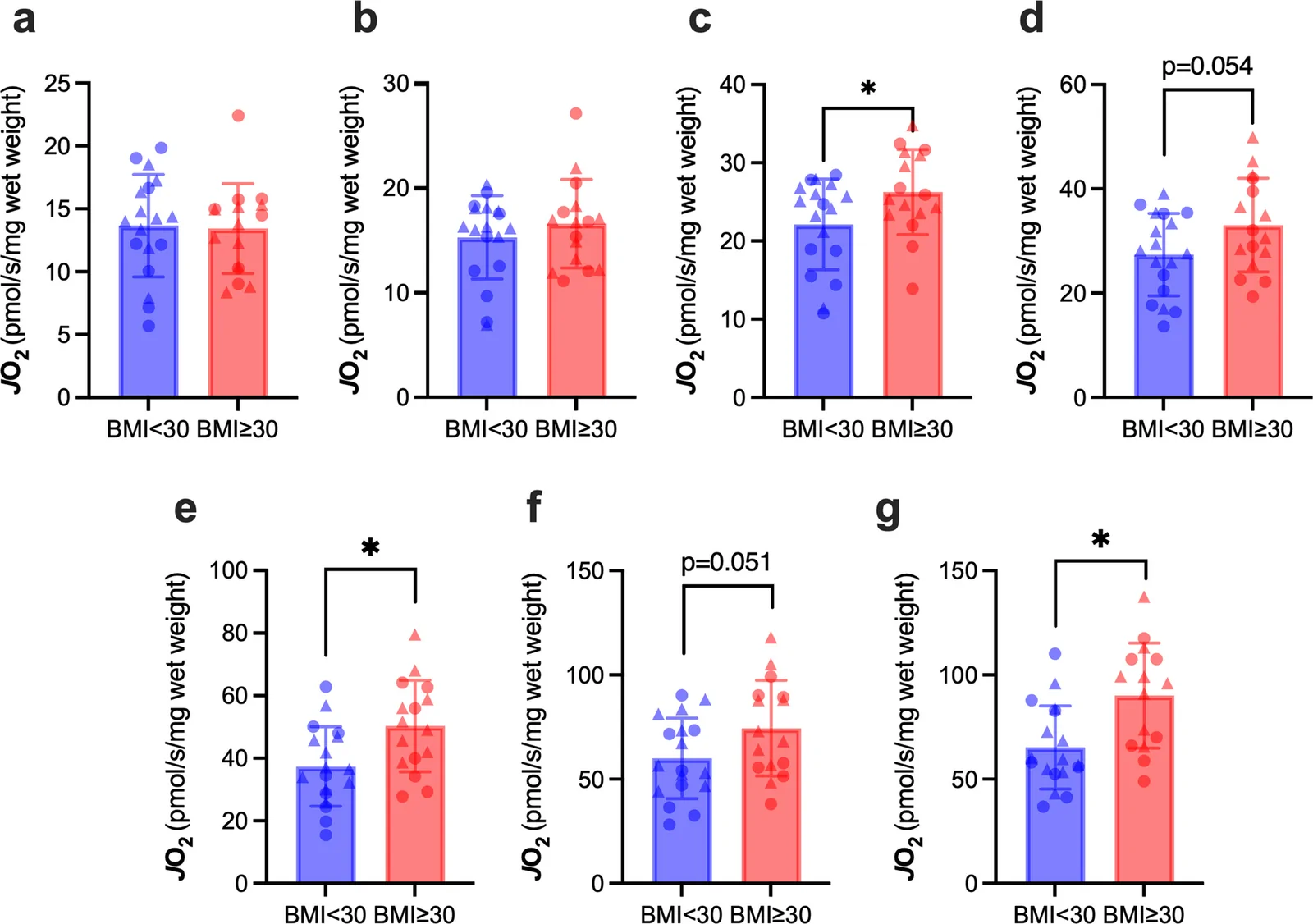
Mitochondrial respiration in permeabilised vastus lateralis muscle fibres in participants without obesity (BMI < 30; *n* = 18) and participants with obesity (BMI ≥ 30; *n* = 16). Mitochondrial respiration was assessed: (**a**) prior to addition of exogenous substrates (routine); (**b**) following addition of the complex I substrates malate, pyruvate and glutamate (leak); following the addition of submaximal (250 µM [c] and 750 µM [d]) and maximal (5000 µM) ADP (**e**); following the addition of the complex II substrate succinate (**f**); and following stepwise titrations of the uncoupler CCCP (**g**; ETS). Data are presented as mean ± SD. Triangles represent female participants and circles represent male participants. Data were analysed using linear models adjusted for sex (*p < 0.05, see text for exact *p* values and effect sizes)

Oxygen consumption rates (*J*O_2_) in permeabilised muscle fibres are shown in Fig. 1. Data from two participants with obesity and one participant without obesity were excluded due to low baseline (routine) values and a lack of increase in respiration with ADP and uncoupler titrations indicative of insufficient mitochondrial functionality for accurate measurements. Routine (Fig. 1a; *p* = 0.813, *d* = 0.1) and leak (Fig. 1b; *p* = 0.541, *d* = 0.2) respiration did not differ between groups. Complex I-supported OXPHOS was higher in participants with obesity in the presence of both submaximal (*p* = 0.044 and *p* = 0.054; both *d* = 0.7) and maximal ADP (*p* = 0.005, *d* = 0.8) concentrations (Fig. 1c–e). Similarly, Complex I + II-supported OXPHOS (Fig. 1f; *p* = 0.051, *d* = 0.7) and electron transfer system capacity (ETS) (Fig. 1g; *p* = 0.011, *d* = 1.0) were higher in those with obesity than in those without.

**Fig. 2:**
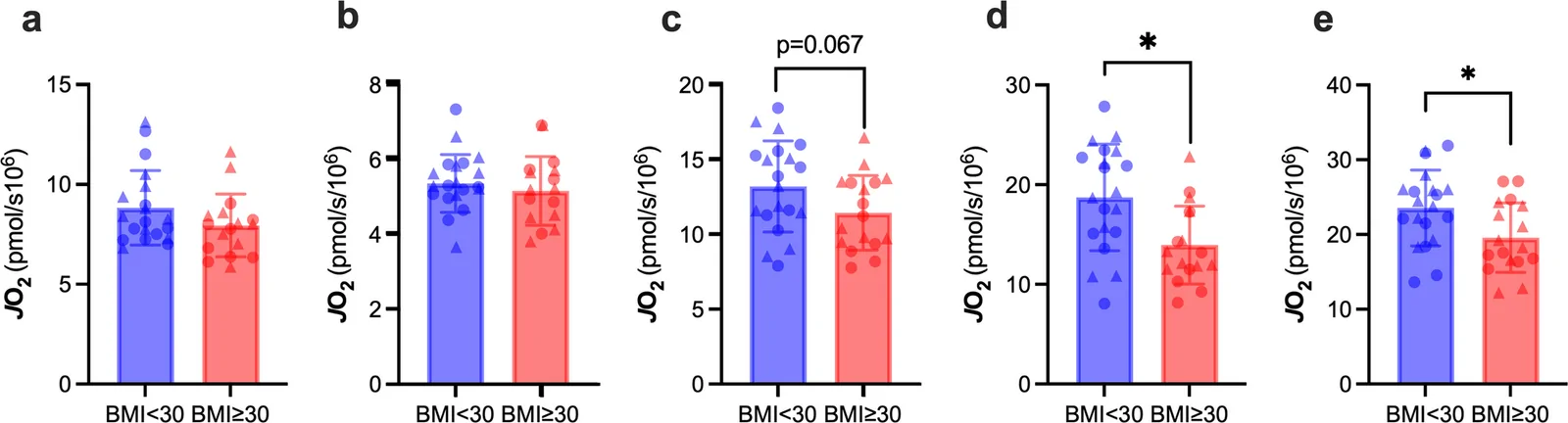
Mitochondrial respiration in isolated CD14 + blood monocytes in participants without obesity (BMI < 30; *n* = 20) and participants with obesity (BMI ≥ 30; *n* = 17). Mitochondrial respiration was assessed: (**a**) prior to addition of exogenous substrates (routine); (**b**) following addition of complex I substrates malate, pyruvate and glutamate (leak); (**c**) following the addition of maximal (2500 µM) ADP; (**d**) following the addition of the complex II substrate succinate (CI + II OXPHOS); and (**e**) following stepwise titrations of the uncoupler CCCP (ETS). Data are presented as mean ± SD. Triangles represent female participants and circles represent male participants. Data were analysed using linear models adjusted for sex (*p < 0.05, see text for exact *p* values and effect size)

Oxygen consumption rate (*J*O_2_) in isolated CD14 + blood monocytes is shown in Fig. 2. Routine (Fig. 2a; *p* = 0.128, *d* = 0.5) and leak (Fig. 2b; *p* = 0.493, *d* = 0.2) respiration did not differ significantly between groups. Contrary to muscle, complex I OXPHOS tended to be lower (Fig. 2c; *p* = 0.067, *d* = 0.6) and complex I + II OXPHOS (Fig. 2d; *p* = 0.005, *d* = 1.0) and ETS (Fig. 2e; *p* = 0.020, *d* = 0.8) were significantly lower in participants with obesity than in those without.

Coupling and pathway control ratios for skeletal muscle and monocytes are shown in Table 2 and Table 3, respectively (definitions and calculations are provided in ESM Table 4). There were no between-group differences in coupling or pathway control ratios in skeletal muscle (*p* > 0.130, *d* = 0.3–0.5). Monocytes from participants with obesity had a higher L/E ratio (the proportion of ETS that is leak respiration) (*p* = 0.043, *d* = 0.7) and a tendency for higher L/P ratio (the proportion of OXPHOS capacity that is leak respiration) (*p* = 0.065, *d* = 0.6), collectively indicative of a greater proportion of respiratory capacity lost to leak respiration. Monocyte P/E ratio (OXPHOS capacity as a proportion of maximal ETS) did not differ between groups (*p* = 0.168, *d* = 0.5). Monocytes from participants with obesity also had higher N/NS (NADH-linked [complex I] pathway contribution to total flux through complex I + II) (*p* < 0.001, *d* = 1.2), indicative of a greater reliance on complex I substrates.

**Table 2:** Coupling and pathway control ratios for skeletal muscle

Ratio	Without obesity (BMI < 30)	With obesity (BMI ≥ 30)
Coupling control ratios		
L/E	0.25 ± 0.08	0.20 ± 0.09
P/E	0.94 ± 0.32	0.85 ± 0.07
L/P	0.27 ± 0.08	0.24 ± 0.10
Pathway control ratios (P-state)		
N/NS	0.62 ± 0.09	0.65 ± 0.06

Values are mean ± SD. See ESM Table 3 and ESM Table 4 for definitions and calculations. P-state, mitochondrial respiratory capacity in ADP-stimulated state

**Table 3:** Coupling and pathway control ratios for monocytes

Ratio	Without obesity (BMI < 30)	With obesity (BMI ≥ 30)
Coupling control ratios		
L/E	0.24 ± 0.06	0.28 ± 0.06*
P/E	0.79 ± 0.15	0.72 ± 0.15
L/P	0.32 ± 0.13	0.40 ± 0.13†
Pathway control ratios (P-state)		
N/NS	0.72 ± 0.08	0.84 ± 0.10*

Values are mean ± SD. See ESM Table 3 and ESM Table 4 for definitions and calculations. *p < 0.05, †*p* < 0.1 vs BMI < 30 (linear models adjusted for sex; see text and/or ESM Table 2 for exact *p* values and effect sizes). P-state, mitochondrial respiratory capacity in ADP-stimulated state

### Muscle citrate synthase activity and mitochondrial protein content

**Fig. 3:**
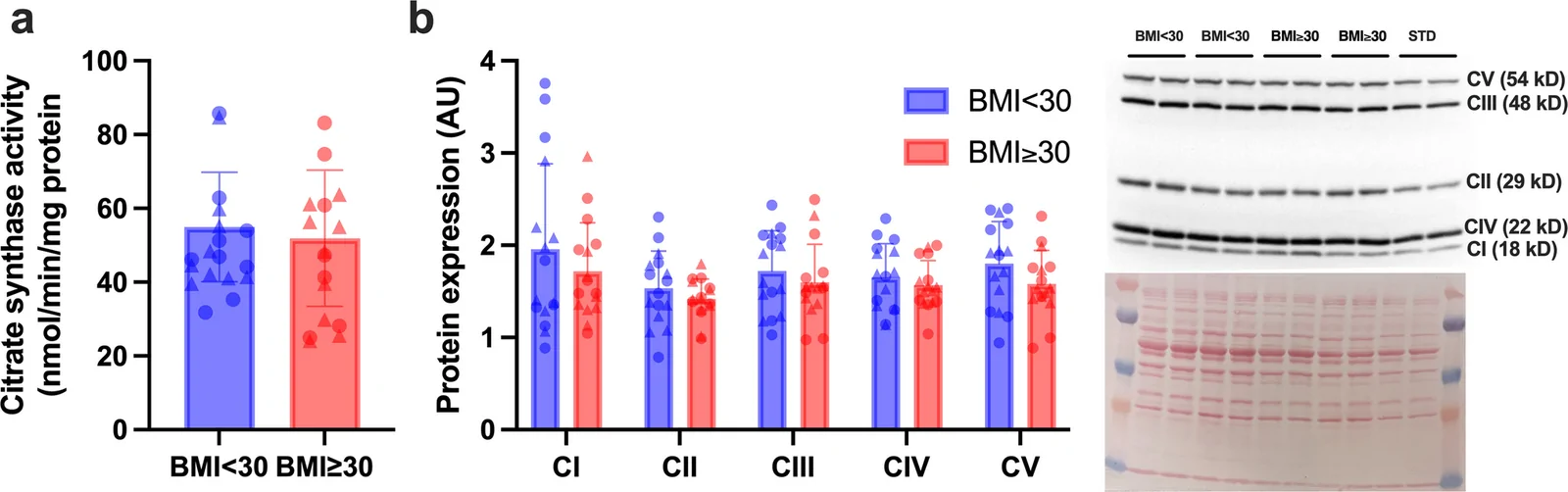
Citrate synthase maximal activity for participants without obesity (BMI < 30; *n* = 18) and with obesity (BMI ≥ 30; *n* = 16) (**a**) and mitochondrial protein content (b; *n* = 16 per group) in skeletal muscle samples. Data are presented as mean ± SD. Triangles represent female participants and circles represent male participants. Data were analysed using linear models adjusted for sex. CI–V, protein subunits of complexes I to V

Citrate synthase maximal activity and the protein content of electron transport chain complex subunits are shown in Fig. 3. Citrate synthase data are from 18 participants without obesity and 16 with obesity and western blot data are from 16 participants per group due to availability of frozen muscle tissue. There was no difference in citrate synthase maximal activity between participants with and without obesity (Fig. 3a; *p* = 0.600, *d* = 0.2). Similarly, there were no between-group differences in any of the muscle mitochondrial proteins measured (Fig. 3b; *p* > 0.148, *d* = 0.2–0.5).

### Blood monocyte phenotype and TLR4 expression

**Fig. 4:**
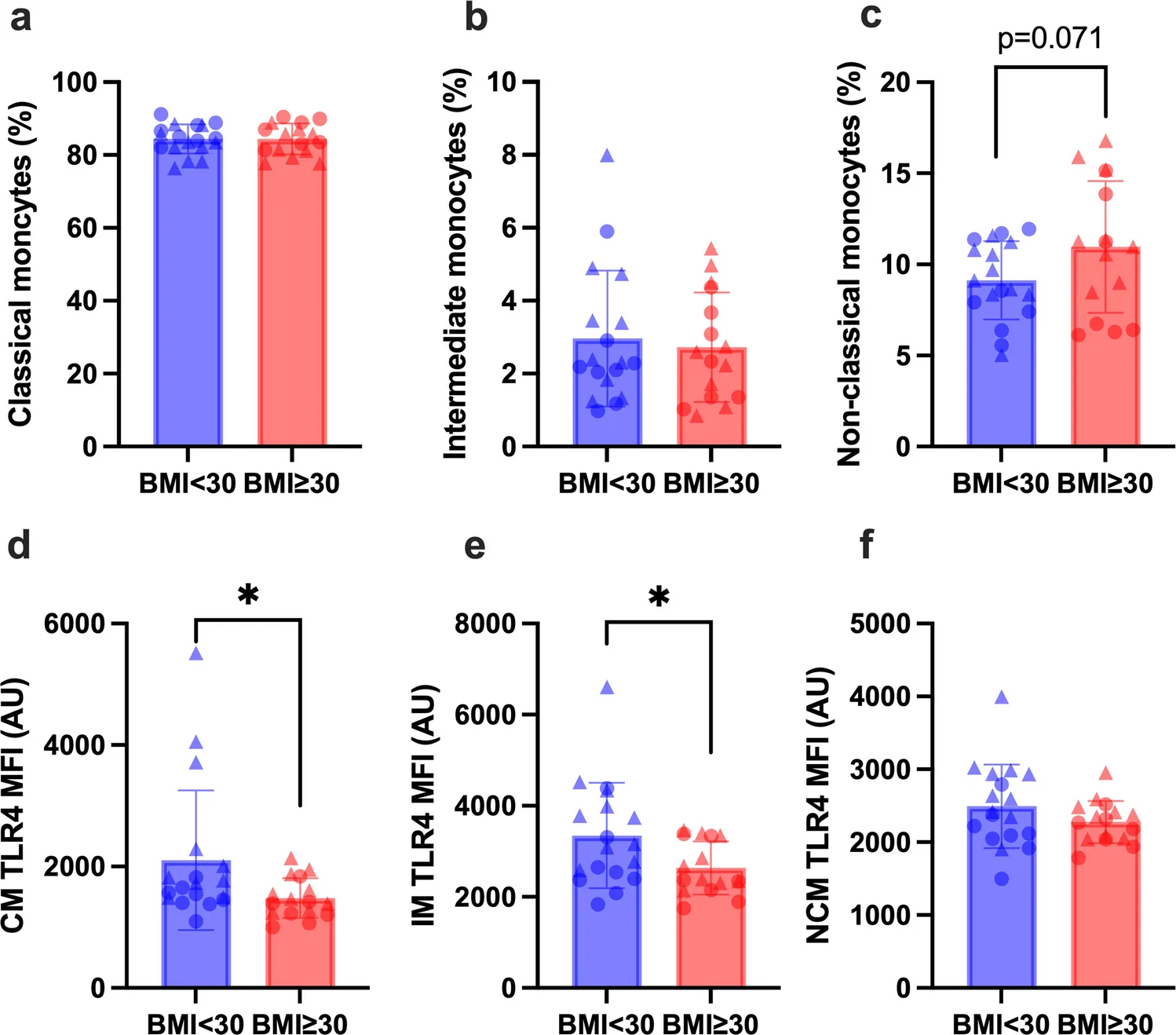
The proportion of classical (**a**), intermediate (**b**) and non-classical (**c**) blood monocytes and TLR4 expression on each subset (d–f) in participants without obesity (BMI < 30; *n* = 18) and participants with obesity (BMI ≥ 30; *n* = 16). Data are presented as mean ± SD. Triangles represent female participants and circles represent male participants. Data were analysed using linear models adjusted for sex (*p < 0.05; see text for exact *p* values and effect size). CM, classical macrophage; IM, intermediate macrophage; NCM, non-classical macrophage

The percentage of each blood monocyte subtype and TLR4 expression on each is shown in Fig. 4. There were no differences in the proportion of classical (Fig. 4a; *p* = 0.990, *d* = 0.0) and intermediate (Fig. 4b; *p* = 0.674, *d* = 0.2) monocytes but participants with obesity tended to have a greater proportion of non-classical monocytes (Fig. 4c; *p* = 0.071, *d* = 0.6) compared with those without obesity. Participants with obesity also had lower expression of TLR4 on both classical (Fig. 4d; *p* = 0.030, *d* = 0.7) and intermediate (Fig. 4e; *p* = 0.023, *d* = 0.8) monocytes. TLR4 expression on non-classical monocytes did not differ between groups (Fig. 4f; *p* = 0.134, *d* = 0.5).

### Blood monocyte polarisation and mitochondrial ROS production ex vivo

**Fig. 5:**
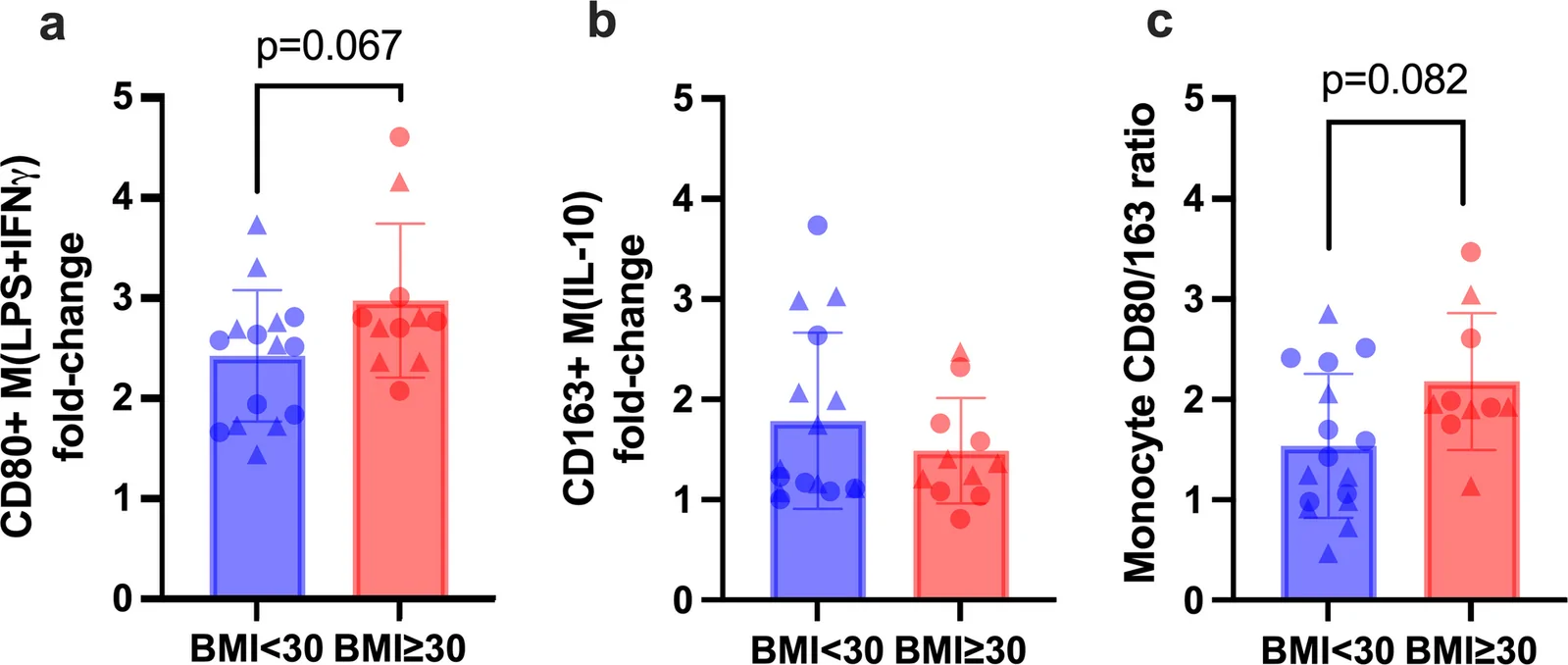
Changes in CD80 expression in M(LPS + IFNɣ) macrophages (**a**) and CD163 expression in M(IL-10) macrophages (**b**) compared with unstimulated cells in participants without obesity (BMI < 30; *n* = 15) and participants with obesity (BMI ≥ 30; *n* = 11), and the ratio of CD80 to CD163 expression in each participant (**c**). Data are presented as mean ± SD. Triangles represent female participants and circles represent male participants. Data were analysed using linear models adjusted for sex (*p < 0.05; see text for exact *p* values and effect size)

Changes in expression of the proinflammatory marker CD80 and the anti-inflammatory marker CD163 in M(LPS + IFNɣ) and M(IL10) monocytes, respectively, compared with unstimulated cells are shown in Fig. 5. Monocyte polarisation experiments were performed on a subset of participants with sufficient monocytes available after high-resolution respirometry. Data are from 15 participants without obesity and 11 participants with obesity. CD80 expression tended to be higher in those with obesity (Fig. 5a; *p* = 0.067, *d* = 0.8) but there was no difference in CD163 expression (Fig. 5b; *p* = 0.594, *d* = 0.2). Participants with obesity also tended to have a higher CD80/CD163 ratio compared with those without obesity (Fig. 5c; *p* = 0.082, *d* = 0.7), indicative of a more proinflammatory monocyte profile.

**Fig. 6:**
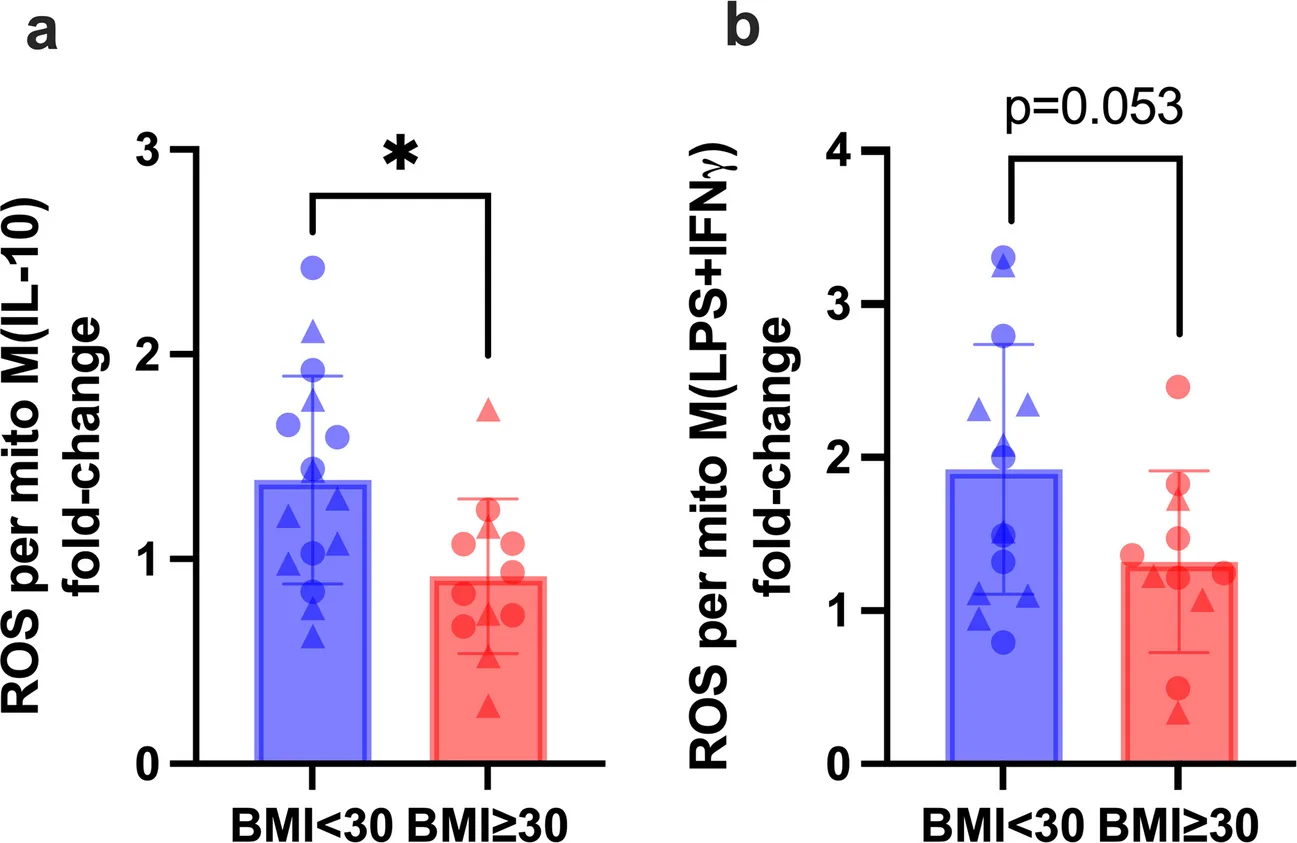
Changes in mitochondria ROS production (MitoSOX Red) normalised to mitochondrial mass (Mito Tracker Green) in M(IL-10) (**a**) and M(LPS + IFNɣ) (**b**) macrophages compared with unstimulated cells from participants without obesity (BMI < 30; *n* = 15) and participants with obesity (BMI ≥ 30; *n* = 11). Data are presented as mean ± SD. Triangles represent female participants and circles represent male participants. Data were analysed using linear models adjusted for sex (*p < 0.05; see text for exact *p* values and effect size)

Changes in mitochondrial ROS production (MitoSOX Red) normalised to mitochondrial mass (Mito Tracker Green) in M(IL-10) and M(LPS + IFNɣ) monocytes, respectively, compared with unstimulated cells are shown in Fig. 6. The increases in mitochondrial ROS production relative to mitochondrial mass were significantly higher in both M(IL-10) (Fig. 6a; *p* = 0.013, *d* = 1.0) and M(LPS + IFNɣ) monocytes (Fig. 6b; *p* = 0.053, *d* = 0.8) from those without obesity compared with those with obesity.

### Correlations between key respiratory, metabolic and immune markers

Correlations between each individual’s monocyte and muscle respiratory states, and between each cell’s or tissue’s respiratory parameters and markers of insulin sensitivity (Matsuda index, HOMA-IR) and systemic inflammation (total white blood cell count) are provided in ESM Table 5. Monocyte and muscle respiratory states were not correlated in either group (*p* > 0.22). Monocyte routine respiration was positively associated with the Matsuda Index across the whole cohort (*r* = 0.47, *p* = 0.007) and inversely associated with HOMA-IR (*r* =− 0.33, *p* = 0.049), with the Matsuda Index association only present in individuals with obesity (*r* = 0.69, *p* = 0.010) when examined within each group. Similar findings were observed for monocyte complex I OXPHOS and the Matsuda Index in participants with obesity (*r* = 0.54, *p* = 0.055). Muscle complex I and complex I + II OXPHOS were inversely associated with the Matsuda Index (*r* =− 0.40, *p* = 0.027 and *r* =− 0.40, *p* = 0.026, respectively) and positively associated with HOMA-IR (*r* = 0.30, *p* = 0.085 and *r* = 0.38, *p* = 0.025) across the whole cohort, with these associations present in individuals without obesity (Matsuda Index: *r* =− 0.48, *p* = 0.046 and *r* =− 0.53, *p* = 0.025; HOMA-IR: *r* = 0.47, *p* = 0.048 for complex I + II OXPHOS) but absent in those with obesity (all *p* > 0.55). No significant associations were observed between any respiratory outcome and total white blood cell count (all *p* > 0.24).

### Correlations between respiratory parameters and participant characteristics

Correlations between respiratory parameters in each cell/tissue and participant characteristics are provided in ESM Table 6. Across the whole cohort, muscle ETS, complex I OXPHOS and complex I + II OXPHOS were positively associated with BMI (*r* = 0.50, *p* = 0.003; *r* = 0.42, *p* = 0.012; and *r* = 0.36, *p* = 0.039, respectively), whereas monocyte complex I + II OXPHOS was inversely associated with BMI (*r* =− 0.40, *p* = 0.013). Muscle leak and complex I + II OXPHOS were inversely associated with age across the whole cohort (*r* =− 0.38, *p* = 0.028 and *r* =− 0.35, *p* = 0.044, respectively), with these associations being stronger and confined to those without obesity (leak − 0.73, *p* = 0.001; complex I + II OXPHOS *r* =− 0.65, *p* = 0.004). Muscle leak respiration was inversely associated with light physical activity across the whole cohort (*r* =− 0.48, *p* = 0.006), and monocyte routine respiration was positively associated with vigorous physical activity in the whole cohort (*r* = 0.34, *p* = 0.046) and in participants without obesity specifically (*r* = 0.60, *p* = 0.011). MVPA was not significantly associated with any respiratory outcome in either group (all *p* > 0.17).

## Discussion

This study compared whole-body immunometabolic outcomes and mitochondrial bioenergetics in skeletal muscle and blood monocytes between young adults with obesity and counterparts without obesity of similar age, sex and physical activity levels. Contrary to previous work, we found that individuals with obesity exhibited higher muscle mitochondrial respiration without alterations in select markers of mitochondrial content compared with their counterparts without obesity. By contrast, blood monocytes from individuals with obesity—precursors to muscle-resident macrophages implicated in muscle insulin resistance—had an attenuated respiratory capacity and lower TLR4 expression despite elevated immune cell counts and non-classical monocyte subsets indicative of a heightened inflammatory state. These in vivo immunometabolic alterations were accompanied by higher proinflammatory marker expression (CD80) and attenuated mitochondrial ROS production in response to ex vivo cytokine stimulation. Our findings highlight divergent bioenergetic alterations in muscle and monocytes from people with obesity coupled with a proinflammatory yet potentially attenuated immunophenotype.

Our findings in muscle differ from previous human studies that report lower muscle oxidative capacity in obesity, especially in older cohorts or those with more severe insulin resistance or type 2 diabetes [4, 6, 9, 13–15]. One explanation for these discrepant findings is that previous work has either involved inactive individuals with obesity or does not appear to adequately account for differences in physical activity when comparing individuals with or without obesity. Notably, in prior studies reporting physical activity or fitness data alongside mitochondrial outcomes, groups were frequently mismatched on these variables, highlighting that reporting a potential confounder and controlling for it are not equivalent and underscoring the value of objectively confirming between-group equivalence in physical activity. This is an important consideration given the known ability of physical activity and exercise to enhance muscle mitochondrial bioenergetic function [20, 21] and that of inactivity to impair it [34]. Another explanation could be the younger age of our cohort with obesity (~ 23 years) compared with previous work involving older participants who had longer exposure to the metabolic environment of obesity [2–4, 14, 15]. In some of these studies, the individuals with obesity were also older than comparator groups without obesity—in some instances, by a decade or more—further confounding interpretation of downstream changes in muscle metabolic function. Our findings combined with this previous work support the notion that impairments in skeletal muscle oxidative capacity observed in obesity may emerge with prolonged metabolic exposure and/or ageing, rather than being an early or intrinsic feature of excess adiposity. This supposition is supported by rodent studies showing that high-fat feeding increases skeletal muscle mitochondrial capacity initially (~ 4 weeks) [11, 35] followed by a subsequent decline with prolonged (~ 4 months) exposure that coincides with insulin resistance [36, 37]. Nevertheless, human studies involving short-term (< 3 weeks) high-fat feeding in healthy adults without obesity [38] and athletes [39] show an impairment in mitochondrial respiratory function, highlighting the need to better understand how excess lipid availability and/or obesity shapes muscle mitochondrial bioenergetics.

Contrary to skeletal muscle, the presence of obesity was associated with a reduction in OXPHOS and ETS in blood monocytes. Monocytes from individuals with obesity also exhibited mitochondrial leakiness and complex I-linked substrate dominance compared with those without obesity, indicating intrinsic bioenergetic inefficiency and metabolic inflexibility. The diminished mitochondrial OXPHOS capacity is in line with the reported shift away from mitochondrial ATP synthesis towards glycolysis under proinflammatory states [30]. Although, to our knowledge, we are the first to report attenuated monocyte mitochondrial respiration using high-resolution respirometry, previous work involving extracellular flux analysis has reported an exaggerated glycolytic activation in activated monocytes from people with obesity [33]. Whereas the attenuated OXPHOS capacity is likely indicative of metabolic rewiring under chronic inflammatory states, the diminished ETS may have consequences for immune responses— known to be compromised in obesity [40]—whereby a limited bioenergetic reserve capacity could constrain effector functions in response to inflammatory stimuli. In line with this supposition, when monocytes from people with obesity were stimulated with LPS and IFNɣ for 24 h ex vivo, the ability of these cells to upregulate mitochondrial ROS production—a mitochondrial-derived signal involved in the activation of the inflammatory and immune responses [30]—was diminished compared with their counterparts without obesity. Notably, although the CD14 + isolation approach used enriches predominantly for classical monocytes (> 80% of circulating monocytes), the observed trend towards higher non-classical monocyte proportions (*p* = 0.071) in those with obesity may have impacted bioenergetic differences, given that monocyte subsets exhibit distinct metabolic profiles. Subset-specific monocyte bioenergetic profiling represents an important direction for future work but will be technically challenging, given the low numbers of circulating non-classical and intermediate monocytes.

Given the established role of muscle–immune cell crosstalk in the contexts of insulin resistance [24], metabolic homeostasis [25] and mitochondrial adaptation [27], we conducted correlation analyses between matched monocyte and muscle respiratory states, and between each tissue's respiratory parameters and whole-body metabolic indices (ESM Table 5). Matched monocyte and muscle respiratory states were not correlated in either group, suggesting that circulating monocyte bioenergetic capacity does not reflect skeletal muscle oxidative capacity within individuals. The lack of association between respiratory parameters suggests that the metabolic environment of obesity has distinct impacts on each cell/tissue—as supported by divergent group-level differences in muscle and monocyte respiration—and that blood-based bioenergetic profiling of monocytes is unlikely to serve as a surrogate marker of muscle mitochondrial function in this context. Nonetheless, monocyte routine respiration was positively associated with the Matsuda Index in the whole cohort (*r* = 0.47), with this relationship being stronger in individuals with obesity (*r* = 0.69). These relationships suggest that preserved bioenergetic capacity in circulating monocytes tracks with better whole-body insulin sensitivity in the context of obesity.

The positive associations between BMI and muscle respiratory parameters and the inverse association between BMI and monocyte OXPHOS capacity are consistent with the divergent tissue-specific bioenergetic alterations observed between groups. The inverse relationship between age and muscle respiratory capacity—particularly in individuals without obesity—supports the idea that previous studies reporting impairments in muscle respiration with obesity may be confounded by the influence of age. Importantly, the absence of significant associations between MVPA and any respiratory outcome in either group provides reassurance that the observed bioenergetic differences are driven by obesity rather than habitual physical activity. Despite these findings, we caution overinterpretation of these exploratory correlational relationships as the study was not explicitly designed nor powered for these analyses. As such, the potential correlations observed in our study (or lack thereof) could be spurious rather than biologically meaningful.

The obesity-associated bioenergetic alterations in monocytes were also accompanied by lower TLR4 expression on blood monocytes in vivo, suggestive of a potentially dysregulated immunophenotype amid a chronic inflammatory state characterised by elevated immune cell counts and expanded non-classical monocyte subsets. Although this lower TLR4 expression challenges the traditional view of obesity-induced inflammation, whereby immune cells retain a heightened activation state [41], it is consistent with chronic TLR4 engagement in the context of metabolic endotoxaemia [42], whereby persistent low-grade LPS and nutrient-derived ligands drive receptor internalisation and degradation [43]. Despite lower TLR4 expression in vivo, monocytes from individuals with obesity expressed higher CD80 levels and had an elevated CD80/CD163 ratio after 24 h LPS/IFNγ stimulation ex vivo—collectively indicative of a more proinflammatory ‘M1-like’ immunophenotype [44]. Previous work shows that IFNγ alone robustly induces CD80 expression on monocytes [45] and that IFNγ signalling is exaggerated in human obesity [46], priming these cells for enhanced M1-like costimulatory responses despite reduced TLR4. Alternatively, we postulate that lower in vivo TLR4 primarily reflects metabolic adaptation to chronic inflammation rather than intrinsic deficiency such that, when stimulated ex vivo with strong proinflammatory cues, monocytes retain (and amplify) their capacity to polarise towards an M1-like state in obesity.

The present study had several notable strengths including the matching of participant physical activity levels, sex and age to isolate the effects of obesity on immunometabolic outcomes. Given that monocyte mitochondrial metabolism was studied in the basal (resting) state to capture in vivo physiology, we did not have sufficient cells to also measure mitochondrial respiratory parameters in response to immune-activating stimuli. Similarly, while the use of high-resolution respirometry to assess bioenergetic function provided insights into additional respiratory parameters that cannot be gleaned from extracellular flux analysis, we recognise that measuring glycolytic metabolism would have provided additional insights into immunometabolic rewiring in obesity. We also acknowledge that factors outside those measured in the study may have contributed to the elevated muscle respiratory capacity observed in participants with obesity, including chronically elevated circulating fatty acids driving higher fat oxidation, potential shifts in fibre type composition towards oxidative type I fibres and normalisation to wet tissue weight, which does not account for potential differences in intramyocellular lipid content, extracellular matrix or water fraction between groups. Future work incorporating intramyocellular lipid quantification would allow direct testing of the lipotoxicity hypothesis, whereby initial adaptive upregulation of fat oxidative capacity may progress towards mitochondrial dysfunction through lipid accumulation over time—a trajectory that may partly explain the discordant mitochondrial findings reported across cohorts with obesity of differing ages and disease duration. Nevertheless, the absence of between-group differences in citrate synthase activity and OXPHOS subunit protein expression (Fig. 3) supports an argument against greater mitochondrial density per unit wet weight as the primary explanation for the elevated respiration observed in individuals with obesity, and normalising to these markers was not pursued given the additional biological noise introduced in small samples and the analytical inconsistency that would arise from applying different normalisation approaches across the two primary tissue outcomes. Participants had similar accelerometry-derived MVPA as contractile activity represents the primary stimulus for skeletal muscle mitochondrial adaptation and therefore provides a more mechanistically direct control for mitochondrial outcomes than cardiorespiratory fitness. Nevertheless, fitness (e.g. maximal oxygen uptake) was not directly measured and cannot be excluded as a contributor to the observed between-group differences in muscle respiratory capacity, and its assessment will be an important addition in future work. Relatedly, as weight-bearing activities impose a greater absolute metabolic load in individuals with higher body mass, equivalent accelerometry-derived MVPA outputs may correspond to higher physical activity energy expenditure in individuals with obesity, potentially contributing to the elevated muscle respiratory capacity observed in this group. We employed a mixed-meal tolerance test as an ecologically valid, integrated assessment of postprandial insulin sensitivity under physiologically relevant conditions (as opposed to an OGTT), although we acknowledge that the mixed-meal tolerance test yields an indirect estimate of insulin sensitivity that is less precise than the gold-standard hyperinsulinaemic–euglycaemic clamp. Lastly, the fluorescent mitochondrial probes used in our study are also known to be highly sensitive to light and temperature; caution is warranted when interpreting these data, especially given the lower sample size for these outcomes.

To our knowledge, this study provides the first demonstration that young adults with obesity exhibit higher skeletal muscle mitochondrial respiration than their counterparts without obesity of similar age, sex and activity levels, without differences in select markers of mitochondrial content. These findings challenge the view that muscle metabolic impairments are an intrinsic feature of excess adiposity and instead corroborate classic rodent work pointing to an early compensatory increase in mitochondrial capacity in the face of an energy surplus. By contrast, circulating blood monocytes from people with obesity display reduced respiratory capacity and a dysregulated immunophenotype in the presence of a systemic inflammatory state. These findings highlight divergent immunometabolic alterations in blood monocytes and skeletal muscle that occur during obesity, and emphasise the importance of tissue-specific contexts when studying mitochondrial profiles in metabolic disease contexts.

## Supplementary Information

Below is the link to the electronic supplementary material.

Supplementary file1 (PDF 964 KB)

## Data Availability

The data underlying this article will be shared upon reasonable request to the corresponding author, consistent with institutional policies and participant consent.

## Abbreviations

ETS: Electron transfer system capacity
LPS: Lipopolysaccharide
mtROS: Mitochondrial reactive oxygen species
MVPA: Moderate-to-vigorous physical activity
OXPHOS: Oxidative phosphorylation
ROS: Reactive oxygen species
TLR4: Toll-like receptor 4

## Code availability

The code used for analyses will be shared upon reasonable request to the corresponding author.

## Declarations

### Authors' relationships and activities

JPL is Chief Scientific Officer for the not-for-profit Institute for Personalised Therapeutic Nutrition. JPL holds founder shares in Metabolic Insights Inc., a for-profit company that developed non-invasive metabolic monitoring devices. All other authors declare that there are no relationships or activities that might bias, or be perceived to bias, their work.
