# Supplementary material for: Immunometabolic alterations in young adults with obesity are accompanied by divergent monocyte and muscle mitochondrial bioenergetic profiles
Source: Metabologia. 2026 Nov 14;2(1):24. doi: 10.1007/s44357-026-00031-5 (PMC13769731; doi:10.1007/s44357-026-00031-5)
Supplement: Supplementary file 1 [file 44357_2026_31_MOESM1_ESM.pdf]

Electronic Supplementary Material (ESM)

METHODS

Skeletal muscle biopsy

Skeletal muscle biopsies were obtained through an incision on the lateral portion of the vastus lateralis (right leg) under superficial local anesthesia (2% lidocaine with epinephrine) using the Bergström muscle biopsy technique modified with manual suction (1). A fresh portion was immediately transferred to BIOPS buffer for HRR (Oroboros O2k) in permeabilized fibers using established substrate–uncoupler–inhibitor titration (SUIT) protocols. The remaining tissue was immediately snap-frozen in liquid nitrogen and stored at -80 for analyses of mitochondrial protein content and citrate synthase (CS) maximal activity.

Intravenous catheterization and mixed meal tolerance testing (MMTT)

An 18-gauge indwelling intravenous catheter was inserted into an antecubital vein for repeated blood collection (BD Nexiva). A fasted blood sample (30-40 mL) was collected into an ethylenediaminetetraacetic acid (EDTA)-coated vacutainer tubes for fasting hematological and metabolic analyses, and monocyte isolation. Participants were then provided with a meal replacement beverage (Ensure Plus Calories: 350 calories, 50 grams of carbohydrates, 11 grams of fat, and 13 grams of protein) to consume within 5 minutes. Following beverage consumption, additional 4 mL blood samples were obtained 15-, 30-, 60-, 90-, and 120-minute post-prandial time-points. Blood was processed immediately for storage of isolated plasma or used for downstream analyses.

Blood processing, hematological analyses, and monocyte isolation

A portion of the baseline blood sample and each subsequent post-prandial blood sample was immediately centrifugated at 2000 × *g* for 10 minutes at 4 °C and isolated plasma was aliquoted and stored at –80 °C for the analysis of plasma insulin and glucose. Complete blood counts were determined in 17 uL of fresh whole blood using an automated hematology analyzer (Beckman Coulter DxH 520). Approximately 100 uL of whole blood was used for the determination of blood monocyte phenotypes using flow cytometry (described below). The remaining whole-blood was used for CD14+ monocyte isolation using an immunomagnetic negative selection kit according to manufacturer instructions (EasySep™ Direct Human Monocyte Isolation Kit, Stemcell Technologies). Isolated monocytes were counted on an automated cell counter (Thermo Fisher Countess 2) and used for high-resolution respirometry or *ex vivo* monocyte polarization as described below.

High resolution respirometry

Mitochondrial respiration in CD14+ monocytes was assessed using 1.5–2 million cells added in duplicate to two Oroboros Oxygraph 2k chambers containing 500 uL of mitochondrial respiration

medium (MiR05). Measurements were made at 37℃ with constant stirring (750 rpm) following air calibration performed before each experiment. Data were recorded in DatLab version 7.4 (Oroboros Instruments). Following the determination of routine respiration in intact cells, monocytes were permeabilized using digitonin (5 ug/mL), followed by the addition of complex I (CI) substrates malate (2 mM), pyruvate (5 mM) and glutamate (10 mM) to assess leak respiration through CI. Subsequently, oxidative phosphorylation capacity (OXPHOS) in the presence of CI substrates was assessed via addition of ADP (2.5 mM). Following the addition of ADP, succinate (10 mM) was added to determine maximal OXPHOS through CI+II. The maximal capacity of the electron transport system (ETS) was then assessed through the stepwise addition of the uncoupler carbonyl cyanide m-chlorophenyl hydrazone (CCCP; 0.5 uM steps) until maximal ETS through CI+II was achieved. Residual oxygen consumption (ROX) was determined following addition of antimycin A (2.5 uM) and subtracted from all respiratory states. Each experiment was completed before the oxygen concentrations reached 50 µM.

Immediately following biopsy, skeletal muscle (~20-30 mg) was placed in ice-cold BIOPS buffer. Fiber bundles (~2–5 mg) were carefully teased apart using sharp surgical tweezers under a dissection microscope. Fiber bundles were transferred into a 12-well culture plate containing 2 mL of freshly prepared saponin solution (50 µg/mL in BIOPS buffer), placed on wet ice, and shaken continuously for 30 minutes. Following permeabilization, fibers were transferred into ice-cold MiR05 buffer and shaken for 10 minutes. The wet weight of muscle fiber bundles was then determined by blotting fibers on Kimwipes until no apparent moisture was seen on the Kimwipe and weighing them using an analytical scale; three measurements were taken, and the average was used. For respiration measurements, 2-5 mg of tissue was added in duplicate Oxygraph 2K chambers containing 2 mL of MiR05, and oxygen concentration was kept between 200–400 µM during the protocol. After assessing routine respiration, leak respiration was assessed following the addition of blebbistatin (5µM) and CI substrates malate (2 mM), pyruvate (5 mM), and glutamate (10 mM). ADP was then titrated at submaximal (250 µM and 750 µM) and saturating (5000 µM) concentrations to determine submaximal and maximal OXPHOS capacity through CI, respectively. Succinate (20 mM) was added hereafter to determine maximal OXPHOS through CI+II. Finally, ETS was determined via the stepwise addition of CCCP (0.5 µM steps). Each experiment was completed before the oxygen concentrations reached 150 µM. Respiratory states and titrations steps are summarized in **ESM Table 3.**

Citrate synthase activity

Citrate synthase (CS) maximal activity (a validated biomarker of mitochondrial content) was measured in previously snap-frozen muscle samples based on the protocol adapted from Jannsen and Boyle (2). Briefly, ~8-10 mg muscle was homogenized using glass-on-glass homogenizer (KIMBLE Dounce tissue grinder set) using 20 strokes then transferred to a 15 mL microcentrifuge tube and centrifugated at 600 g at 4ºC. The supernatant was collected and diluted 10:1 in Sample Lysis Buffer (210 mM mannitol, 70 mM sucrose, 5 mM HEPES, 1 mM EGTA, pH 7.2, adjusted with KOH). The protein concentration of the diluted muscle homogenate supernatant was measured by Pierce™ BCA Protein Assay Kits according to manufacturer's instructions. 10 µL of muscle homogenate was transferred to 96-well plates (Corning® 96 Well Clear Polystyrene Microplate

Flatbottom plates), containing 186 µL of Assay Solution (50 mM Kpi Buffer, 100 µM (5,5-dithio-bis-(2-nitrobenzoic acid) and 115 µM Acetyl-CoA. The maximal activity of CS was then measured by assessing rate of thionitrobenzoate appearance based on the change of absorbance for 165 seconds at 415nm on a iMark™ Microplate Absorbance Reader following the addition of 4 µL of 5mM oxaloacetate. CS maximal activity was corrected to the mg protein from the diluted muscle homogenate.

Western blotting

Muscle (~20 mg) was homogenized in RIPA buffer (Thermo Fisher) and mechanically homogenized (Omni Tissue Homogenizer). Using sample buffer (Novex Tris-Glycine SDS Sample Buffer (2X)), the samples were then solubilized and heated to 85°C for 5 min. Precast 12% polyacrylamide gels were loaded with equal amounts of protein (10 µg for each sample and internal loading control) and separated by SDS-PAGE for 40 minutes at 225 volts (Novex Tris-Glycine SDS Running Buffer). The protein transfer was performed using polyvinylidene difluoride membrane (PVDF) via wet transfer for 60 min at 20 volts (Novex Tris Glycine Transfer Buffer). Following the transfer and three washes for 3 minutes each in PBS-T, membranes were blocked at room temperature by incubating in 5% BSA with 0.1 Tween-20 (PBS-T) on a rocking platform. An overnight incubation of the membranes was performed on a rocking platform at 4°C with primary antibodies in 5% BSA with PBS-T. Commercially available antibodies were used (OxPhos Human WB Antibody Cocktail, 1:1000 dilution; Thermo Fisher Scientific, Cat#45-8199) for Complex I subunit [NDUFB8], Complex II subunit 30kDa [SDHB], Complex III subunit Core 2 [UQCRC2], Complex IV subunit II [MTCO2], and ATP synthase subunit alpha. Following the overnight incubation, excess primary antibodies were removed by washing the membranes three times for 3 min each in PBS-T. The secondary antibody goat anti-mouse IgG (lot 3229484, ThermoFisher) was diluted (1:10,000 PBS-T 0.1%) and membranes were incubated for 1 hour at room temperature. To remove excess secondary antibodies, the membranes were washed three times with PBS-T for 3 minutes. Membranes were incubated in SuperSignal™ West Dura Extended Duration Substrate for 5 minutes at room temperature before visualizing using an iBright CL750 Imaging System (ThermoFisher). Band intensities were quantified and corrected using the internal control using the iBright software. Ponceau Red staining was used as a loading/transfer control to ensure equal loading across samples/groups – in line with recommendations for its use over conventional housekeeping proteins given their susceptibility to variation with experimental conditions, physiological interventions, and tissue composition, and for providing a broader linear dynamic range while avoiding saturation artifacts inherent to single-protein controls (3).

Blood monocyte phenotype

Whole blood was conjugated to the following fluorophore-labelled monoclonal antibodies: anti-CD14 (1:50 VioBlue, 130-110-524, Miltenyi Biotec), anti-CD16 (1:25 PE-Vio770, 130-113-394, Miltenyi Biotec), and anti-CD284 (also known as TLR4; 1:50 APC, 130-100-150, Miltenyi Biotec), and incubated in the dark at 4°C for 10 minutes. Cells were then lysed with 1X red blood cell lysis solution (Miltenyi Biotec), incubated for another 10 minutes in the dark, and read immediately on a flow cytometer (Cytoflex S; Beckman Coulter). Gating strategy was established via

fluorescence minus one (FMO) and compensation, with gates around positive cell populations. Cells were initially gated on monocyte population by forward and side scatter, with monocyte populations confirmed through gating on a CD14+ population (10,000 events collected per experiment). This population was further gated on CD16+ and CD284+ cells. Classical (CD14++, CD16−), intermediate (CD14++, CD16+), and non-classical (CD14+, CD16++) monocytes were determined based on relative expressions of CD14 and CD16 (relative % expression). Median fluorescence intensity (MFI) was used to determine TLR4 expression on each subset based on CD284 staining.

Monocyte polarization and mitochondrial ROS production

Monocytes were polarized towards a pro- ("M1-like") or anti-inflammatory ("M2-like") phenotype as described previously (4). Briefly, CD14+ monocytes were plated in a 24-well culture plate at a density of ~250-300,000 cells per well in a final volume of 500 µL. Cells were stimulated with lipopolysaccharide (LPS; 50 ng/mL) and interferon gamma (IFNɣ; 20 ng/mL) to promote a pro-inflammatory phenotype [M(LPS+IFNɣ)] or with interleukin-10 (20 ng/mL) to promote an anti-inflammatory phenotype [M(IL-10)]. Cells were cultured in RPMI supplemented with 5 mM glucose, 10% fetal bovine serum and 20 mM HEPES in a humidified incubator (37℃, 5% CO_2) for 24 hrs. Hereafter, cells were removed for surface staining with pro- (CD80; CAT# 130-123-253, Miltenyi Biotec) and anti- (CD163; 130-112-290, Miltenyi Biotech) inflammatory markers. Cells were stained for 10 min in the dark and analyzed immediately on a flow cytometer (Cytoflex S, Beckman Coulter; as described above for blood monocyte phenotype) with CD80 and CD163 MFI determined on 5000 CD14+ monocytes. Cells from replicate wells were stained with Mito Tracker Green (50 nM) and MitoSOX Red (5 µM) and incubated in the dark at 37℃ for 30 minutes with anti-CD14 antibody (1:100 VioBlue, 130-110-524, Miltenyi Biotec) added during the last 10 minutes of incubation. The MFI of Mito Tracker Green and MitoSOX Red staining in 5000 CD14+ cells was quantified on a flow cytometer (Cytoflex S, Beckman Coulter).

Plasma insulin and glucose

Insulin and glucose were measured in duplicate from previously frozen isolated plasma using the CrystalChem Human Insulin ELISA Kit (Cat#90096) and CrystalChem Glucose Assay Kit (Cat#81697). Fasting insulin resistance (IR) was estimated based on homeostasis model assessment (HOMA)-IR using the following formula: HOMA-IR = [fasting glucose (mmol/L) × fasting insulin (µU/mL)] / 22.5 (5). Insulin sensitivity was calculated using the Matsuda index with the following formula: 10,000/√[fasting glucose (mmol/l) × fasting insulin (pmol/l)] × [mean glucose(mmol/l) × mean insulin(pmol/l) during MMTT] (6).

Accelerometer-derived physical activity

Participants were provided with an ActiGraph GT3X+ (Pensacola, FL) accelerometer to wear on the waist for 7 consecutive days. Participants were instructed to only remove the device during water-based activities and while sleeping. Devices were initialized to record raw tri-axial acceleration data at a frequency of 30Hz with a dynamic range of ±8g. Upon retrieval, raw

acceleration .csv files were exported using the ActiLife® software (version 6.13.3) and processed using the open-source R package GGIR (version 4.3.2) using default package arguments for signal processing. These include: 1) auto-calibration using local gravity as a reference; 2) calculation of Euclidean Norm minus 1g (ENMO) with data averaged over 5 second epochs and expressed in milli-gravitational units (mg); 3) detection of non-wear time; and 4) detection of abnormally sustained values. Thresholds for interpreting light (>50mg), moderate (>70mg) and vigorous (>260mg) activity were set using previously validated cut-points. Participants with fewer than 3 valid wear days (>10 hours/day) were excluded from analysis. To minimize misclassifications of activity intensity inherent to cut-point approaches (7), MVPA was quantified using bouts ≥10 minute to better reflect sustained, purposeful activity (8).

SUPPLEMENTARY FIGURES & TABLES

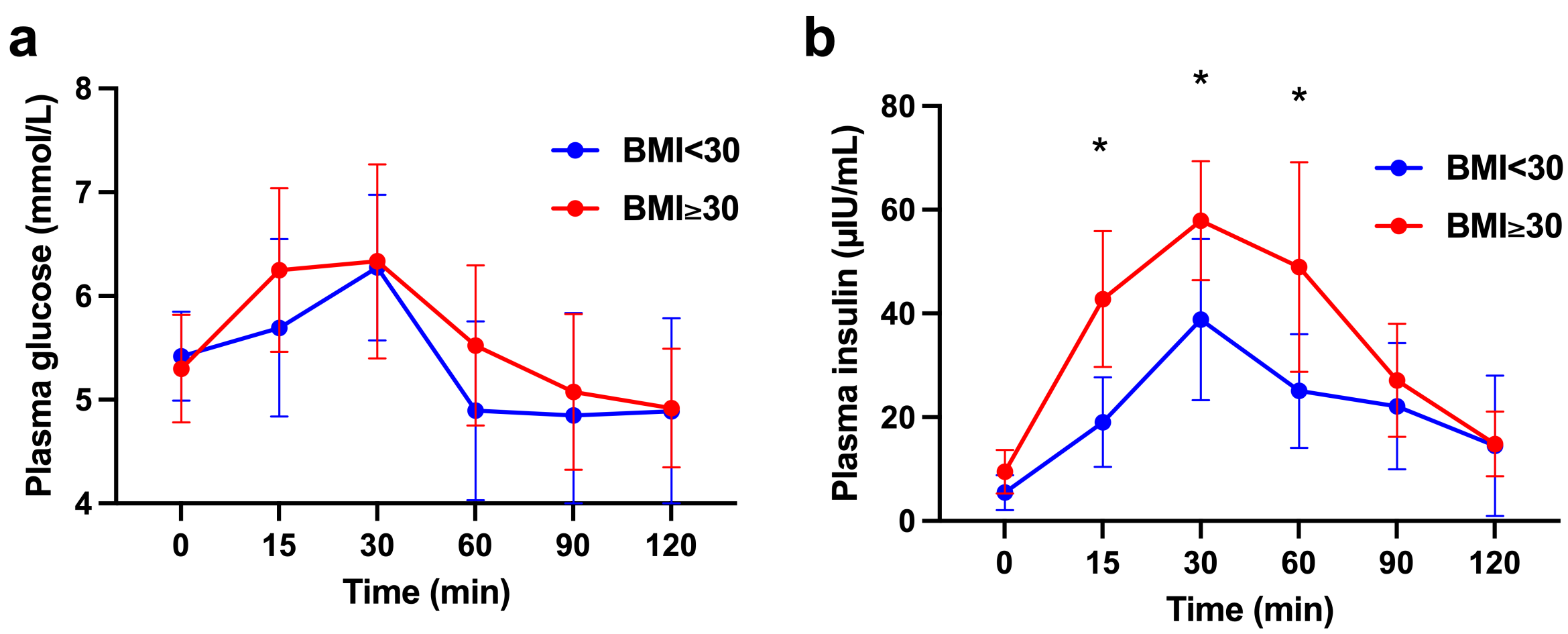

ESM Figure 1. Plasma glucose (A) and insulin (B) responses to the mixed meal tolerance test in BMI<30 (*n*=19) and BMI≥30 (*n*=17). Data were analyzed using linear mixed effects analyses adjusted for sex with group and time as fixed effects and participant as random effect. Bonferroni post-hoc testing was used to interrogate significant main effects and interactions. Data are presented as mean ± SD. A main effect of group, time, and an interaction effect was observed for plasma insulin (all p<0,001). A significant main effect of time was observed for plasma glucose (p<0.001). * denotes significant between-group differences at the indicated timepoints (p<0.05).

ESM table 1. Summary of sample sizes analyzed for each outcome along with reasons for missing data.

Outcome (Figure #)	BMI<30 (*n*)	BMI≥30 (*n*)	Reason for missing or excluded data
Muscle respiration (1)	18	16	Low baseline (routine) values; lack of increase in respiration with ADP and uncoupler titrations indicative of insufficient mitochondrial functionality
Monocyte respiration (2)	20	17	-
Muscle CS activity (3A)	18	16	Lack of available frozen tissue
Muscle OXPHOS protein (3B)	16	16	Lack of available frozen tissue
Blood monocyte subsets (4)	18	16	2 outliers in BMI<30 and 1 outlier in BMI≥30 were removed from analyses

Monocyte CD80/163 expression (5)	15	11	Lack of available monocytes for culture
Monocyte ROS production (6)	15	11	Lack of available monocytes for culture

ESM Table 2. Estimated marginal means [95% confidence intervals] and p-values for all outcomes derived from linear models adjusted for sex.

**Outcome**	**BMI<30**	**BMI≥30**	**P value**
Age (years)	22.8 [20.4, 25.1]	22.9 [20.4, 25.3]	0.934
Sex (F/M)	10/10	9/8	NA
Body mass (kg)	64.5 [60, 70]	101.4 [96, 107]	<0.001
BMI (kg/m^2)	21.8 [20.6, 23.0]	33.0 [31.8, 34.2]	<0.001
Body Fat%	19.5 [17.2, 21.9]	37.4 [34.8, 40.0]	<0.001
Visceral Fat Area (cm^3)	52 [37, 66]	181 [166, 197]	<0.001
Waist circumference (inches)	29.1 [27.8, 30.4]	42.0 [40.6, 43.4]	<0.001
Systolic blood pressure (mmHg)	118 [113, 123]	123 [117, 128]	0.218
Diastolic blood pressure (mmHg)	69.1 [64.5, 73.7]	72.2 [67.5, 76.8]	0.349
Fasting Insulin (µIU/mL)	5.5 [3.8, 7.2]	9.5 [7.7, 11.4]	<0.001
Fasting Glucose (mmol/L)	5.4 [5.2, 5.6]	5.3 [5.1, 5.5]	0.413
Inactivity (min)	953 [869, 1037]	931 [847, 1015]	0.709
Daily light physical activity (min)	77 [53, 101]	83 [59, 107]	0.719
Daily moderate physical activity (min)	44 [31, 57]	50 [37, 63]	0.485
Daily vigorous physical activity (min)	1 [0, 2]	1 [0, 2]	0.833
Daily MVPA Bouts >10 min (min)	16 [9, 22]	13 [6, 21]	0.647
HOMA-IR	1.3 [0.9, 1.8]	2.3 [1.8, 2.7]	0.006
Matsuda Index	11.2 [9.3, 13.0]	6.6 [4.3, 8.8]	0.003
Total white blood cells (x10^3/uL)	5.3 [4.8, 5.9]	6.4 [5.8, 7.0]	0.011
Lymphocytes (x10^3/uL)	1.7 [1.5, 2.0]	2.1 [1.9, 2.3]	0.032
Monocytes (x10^3/uL)	0.43 [0.37, 0.48]	0.46 [0.40, 0.51]	0.501
Neutrophils (x10^3/uL)	3.0 [2.5, 3.5]	3.7 [3.2, 4.2]	0.046
Eosinophils (x10^3/uL)	0.14 [0.10, 0.18]	0.16 [0.12, 0.21]	0.507
Basophils (x10^3/uL)	0.02 [0.01, 0.02]	0.02 [0.01, 0.02]	0.939
Red blood cells (x10^6/uL)	4.71 [4.55, 4.88]	4.77 [4.60, 4.94]	0.623
Hemoglobin (g/dL)	14.6 [14.1, 15.1]	14.4 [13.9, 14.9]	0.526
Hematocrit (%)	42.2 [41.1, 43.3]	42.3 [41.1, 43.3]	0.946
Platelets (x10^3/uL)	247 [221, 272]	265 [238, 292]	0.327
Muscle routine (pmol/sec/mg)	13.7 (11.8, 15.6)	13.3 (11.2, 15.4)	0.813
Muscle leak (pmol/sec/mg)	15.3 (13.3, 17.2)	16.1 (14.0, 18.2)	0.541
Muscle CI (0.25) (pmol/sec/mg)	21.9 (19.3, 24.6)	25.9 (23.1, 28.7)	0.044
Muscle CI (0.75) (pmol/sec/mg)	27.1 (23.2, 31)	32.7 (28.5, 36.8)	0.054
Muscle CI (5.0) (pmol/sec/mg)	37.0 (30.1, 43.9)	48.5 (41.2, 55.8)	0.026
Muscle CI+CII (pmol/sec/mg)	59.5 (49.5, 69.4)	73.9 (63.3, 84.5)	0.051
Muscle ETS (pmol/sec/mg)	65.1 (53.7, 76.4)	87.3 (75.3, 99.3)	0.010
Monocyte routine (pmol/sec/million cells)	8.8 (8.0, 9.6)	7.9 (7.1, 8.8)	0.128
Monocyte leak (pmol/sec/million cells)	5.4 (5.0, 5.8)	5.1 (4.8, 5.6)	0.493
Monocyte CI (pmol/sec/million cells)	13.2 (11.9, 14.5)	11.4 (10.0, 12.8)	0.067
Monocyte CI+CII (pmol/sec/million cells)	18.7 (16.6, 20.9)	13.9 (11.6, 16.3)	0.005
Monocyte ETS (pmol/sec/million cells)	23.6 (21.3, 25.8)	19.6 (17.1, 22.0)	0.020
Muscle L/E ratio	0.25 (0.21, 0.29)	0.20 (0.16, 0.25)	0.131
Muscle P/E ratio	0.94 (0.82, 1.1)	0.85 (0.73, 0.97)	0.268
Muscle L/P ratio	0.27 (0.23, 0.32)	0.24 (0.19, 0.29)	0.318
Muscle N/NS ratio	0.63 (0.59, 0.66)	0.66 (0.62, 0.70)	0.293

Monocyte L/E ratio	0.24 (0.21, 0.26)	0.28 (0.25, 0.31)	0.043
Monocyte P/E ratio	0.79 (0.72, 0.86)	0.72 (0.65, 0.80)	0.168
Monocyte L/P ratio	0.32 (0.26, 0.37)	0.40 (0.34, 0.46)	0.065
Monocyte N/NS ratio	0.72 (0.68, 0.77)	0.84 (0.79, 0.88)	<0.001
CS activity (nmol/min/mg protein)	55.0 (46.9, 63.1)	52.1 (43.5, 60.7)	0.599
CI.NDUFB8.18kD (AU)	2.0 (1.6, 2.4)	1.7 (1.3, 2.1)	0.379
CII.SDHB.29kD (AU)	1.5 (1.4, 1.7)	1.4 (1.3, 1.6)	0.316
CIII.UQCRC2.48kD (AU)	1.7 (1.5, 1.9)	1.6 (1.4, 1.8)	0.423
CIV.COX.II.22kD (AU)	1.7 (1.5, 1.8)	1.6 (1.4, 1.7)	0.443
CV.ATP5A.54kD (AU)	1.8 (1.6, 2.0)	1.6 (1.4, 1.8)	0.148
Classical monocytes (%)	84.5 (82.7, 86.4)	84.6 (82.7, 86.5)	0.990
Intermediate monocytes (%)	2.9 (2.1, 3.7)	2.7 (1.8, 3.5)	0.674
Non-classical monocytes (%)	9.0 (7.7, 10.4)	10.9 (9.4, 12.3)	0.071
CM TLR4 (AU)	2061 (1677, 2446)	1434 (1026, 1842)	0.030
IM TLR4 (AU)	3302 (2887, 3717)	2587 (2147, 3028)	0.023
NCM TLR4 (AU)	2468 (2271, 2664)	2249 (2040, 2458)	0.134
CD80 M(LPS+IFNg) (fold-change)	2.4 (2.0, 2.8)	2.9 (2.5, 3.4)	0.067
CD163 M(IL-10) (fold-change)	1.6 (1.3, 1.9)	1.5 (1.1, 1.9)	0.594
CD80/163 ratio	1.7 (1.3, 2.1)	2.2 (1.7, 2.7)	0.082
ROS per mito M(IL-10) (fold-change)	1.4 (1.2, 1.6)	0.92 (0.64, 1.2)	0.013
ROS per mito M(LPS+IFNg) (fold-change)	1.9 (1.5, 2.3)	1.3 (0.85, 1.8)	0.053
Note: F, female; M, male; BMI, body mass index; MVPA, moderate-vigorous physical activity.			

ESM table 3. Titrations and respiratory states.

**Titration step**	**State**	**Flux**	**Pathway**
Malate + Pyruvate + Glutamate	N-LEAK	$J_{N,L}$	N (CI-linked)
+ ADP	N-P	$J_{N,P}$	N (CI-linked, OXPHOS)
+ Succinate	NS-P	$J_{NS,P}$	NS (CI&II-linked, OXPHOS)
+ CCCP	NS-ET	$E(J_{NS,E})$	NS (maximal ET capacity)

ESM table 4. Calculation and interpretation of coupling and pathway control ratios.

**Ratio**	**Formula**	**Interpretation**
L/E	$J_{N,L}/E$	Proportion of electron transfer capacity (E) that is leak respiration (L) in the absence of ADP. Lower values indicate tighter coupling.
P/E	$J_{NS,P}/E$	OXPHOS capacity (P, with ADP) as a proportion of uncoupled ET capacity (E). Values near 1.0 show phosphorylation system matches or exceeds ET capacity.
L/P	$J_{N,L}/J_{NS,P}$	Proportion of OXPHOS flux (P) that is leak respiration (L). Lower values reflect higher respiratory control.
N/P or N/NS	$J_{N,P}/J_{NS,P}$	NADH-linked (CI) pathway contribution to total flux through CI+II (NS substrates), measured in the same coupling state (P).

ESM table 5. Pearson correlation coefficients between respiratory outcomes and select metabolic/inflammatory indices.

Variable 1	Variable 2	All participants			BMI<30			BMI≥30		
		r	*p*	*n*	*r*	*p*	*n*	*r*	*p*	*n*
**Monocyte × muscle respiration**										
Mono Routine	Muscle Routine	-0.09	0.626	33	-0.15	0.555	18	-0.02	0.932	15
Mono Leak	Muscle Leak	-0.14	0.430	33	-0.05	0.843	18	-0.29	0.295	15
Mono CI OXPHOS	Muscle CI OXPHOS	-0.07	0.679	34	-0.17	0.512	18	0.32	0.223	16
Mono CI+II OXPHOS	Muscle CI+II OXPHOS	-0.15	0.397	34	-0.17	0.508	18	0.27	0.306	16
Mono ETS	Muscle ETS	-0.19	0.279	34	-0.19	0.448	18	0.23	0.389	16
**Monocyte respiration × whole-body metabolic and inflammatory outcomes**										
Mono Routine	HOMA-IR	-0.33	**0.049***	37	-0.23	0.324	20	-0.27	0.286	17
Mono Routine	Matsuda Index	0.47	**0.007***	32	0.38	0.107	19	0.69	**0.010***	13
Mono Routine	Total WBC	-0.01	0.967	37	0.06	0.799	20	0.20	0.441	17
Mono Leak	HOMA-IR	-0.14	0.413	36	-0.30	0.191	20	0.09	0.731	16
Mono Leak	Matsuda Index	0.22	0.220	32	0.34	0.149	19	-0.09	0.767	13
Mono Leak	Total WBC	-0.16	0.359	36	-0.03	0.909	20	-0.20	0.462	16
Mono CI OXPHOS	HOMA-IR	-0.25	0.135	37	-0.10	0.677	20	-0.19	0.476	17
Mono CI OXPHOS	Matsuda Index	0.35	**0.051#**	32	0.12	0.619	19	0.54	**0.055#**	13
Mono CI OXPHOS	Total WBC	-0.02	0.911	37	0.08	0.736	20	0.22	0.405	17
Mono CI+II OXPHOS	HOMA-IR	-0.23	0.168	37	-0.03	0.893	20	-0.05	0.837	17
Mono CI+II OXPHOS	Matsuda Index	0.33	**0.063#**	32	0.09	0.726	19	0.24	0.420	13
Mono CI+II OXPHOS	Total WBC	-0.08	0.642	37	0.09	0.698	20	0.26	0.314	17
Mono ETS	HOMA-IR	-0.09	0.610	37	-0.07	0.765	20	0.28	0.277	17
Mono ETS	Matsuda Index	0.29	0.106	32	0.12	0.612	19	-0.09	0.760	13
Mono ETS	Total WBC	-0.06	0.738	37	0.20	0.408	20	0.08	0.773	17
**Muscle respiration × whole-body metabolic and inflammatory outcomes**										
Muscle Routine	HOMA-IR	0.17	0.346	33	0.11	0.667	18	0.31	0.263	15
Muscle Routine	Matsuda Index	0.08	0.673	31	0.03	0.916	18	-0.09	0.781	13
Muscle Routine	Total WBC	-0.05	0.768	33	-0.11	0.669	18	0.05	0.868	15
Muscle Leak	HOMA-IR	0.28	0.112	34	0.25	0.321	18	0.27	0.319	16
Muscle Leak	Matsuda Index	-0.21	0.266	31	-0.31	0.214	18	0.02	0.957	13
Muscle Leak	Total WBC	0.02	0.899	34	-0.03	0.911	18	-0.03	0.922	16
Muscle CI OXPHOS	HOMA-IR	0.30	**0.085#**	34	0.40	0.104	18	0.01	0.979	16
Muscle CI OXPHOS	Matsuda Index	-0.40	**0.027***	31	-0.48	**0.046***	18	0.18	0.553	13
Muscle CI OXPHOS	Total WBC	0.17	0.336	34	-0.25	0.317	18	0.23	0.396	16
Muscle CI+II OXPHOS	HOMA-IR	0.38	**0.025***	34	0.47	**0.048***	18	0.14	0.597	16
Muscle CI+II OXPHOS	Matsuda Index	-0.40	**0.026***	31	-0.53	**0.025***	18	0.16	0.597	13
Muscle CI+II OXPHOS	Total WBC	0.11	0.544	34	-0.26	0.304	18	0.15	0.574	16
Muscle ETS	HOMA-IR	0.29	**0.094#**	34	0.11	0.657	18	0.17	0.528	16
Muscle ETS	Matsuda Index	-0.28	0.123	31	-0.18	0.475	18	0.15	0.636	13

Muscle ETS	Total WBC	0.16	0.360	34	-0.29	0.238	18	0.17	0.521	16
Note: * p <0.05, # p<0.1										

ESM table 6. Pearson correlation coefficients between respiratory outcomes and participant characteristics.

Variable 1	Variable 2	All participants			BMI<30			BMI≥30		
		r	***p***	***n***	***r***	***p***	***n***	***r***	***p***	***n***
**Monocyte respiration × participant characteristics**										
Mono Routine	Age (years)	0.07	0.684	37	0.11	0.648	20	0.05	0.852	17
Mono Routine	BMI (kg/m²)	-0.25	0.134	37	-0.04	0.860	20	-0.07	0.805	17
Mono Routine	MVPA (min/day)	0.02	0.927	34	0.17	0.518	17	-0.25	0.337	17
Mono Routine	Moderate PA (min/day)	-0.12	0.481	34	-0.02	0.942	17	-0.21	0.424	17
Mono Routine	Vigorous PA (min/day)	**0.34**	**0.046***	34	**0.60**	**0.011***	17	-0.01	0.975	17
Mono Routine	Light PA (min/day)	0.05	0.772	34	0.30	0.236	17	-0.16	0.541	17
Mono Routine	Inactivity (min/day)	-0.10	0.567	34	-0.39	0.125	17	0.14	0.606	17
Mono Leak	Age (years)	0.17	0.328	36	0.33	0.161	20	0.05	0.859	16
Mono Leak	BMI (kg/m²)	-0.07	0.678	36	0.11	0.640	20	0.08	0.770	16
Mono Leak	MVPA (min/day)	-0.28	0.108	33	-0.39	0.125	17	-0.21	0.428	16
Mono Leak	Moderate PA (min/day)	**-0.37**	**0.036***	33	**-0.41**	**0.097#**	17	-0.30	0.251	16
Mono Leak	Vigorous PA (min/day)	-0.02	0.910	33	-0.15	0.576	17	0.08	0.763	16
Mono Leak	Light PA (min/day)	-0.10	0.580	33	-0.01	0.976	17	-0.14	0.613	16
Mono Leak	Inactivity (min/day)	0.14	0.445	33	-0.27	0.299	17	0.41	0.116	16
Mono CI OXPHOS	Age (years)	0.04	0.810	37	0.26	0.276	20	-0.18	0.479	17
Mono CI OXPHOS	BMI (kg/m²)	-0.27	0.106	37	0.03	0.886	20	0.03	0.920	17
Mono CI OXPHOS	MVPA (min/day)	-0.26	0.142	34	-0.25	0.342	17	-0.33	0.195	17
Mono CI OXPHOS	Moderate PA (min/day)	-0.28	0.112	34	-0.34	0.178	17	-0.10	0.690	17
Mono CI OXPHOS	Vigorous PA (min/day)	0.05	0.786	34	0.03	0.903	17	0.05	0.845	17
Mono CI OXPHOS	Light PA (min/day)	0.08	0.648	34	0.24	0.356	17	-0.05	0.845	17
Mono CI OXPHOS	Inactivity (min/day)	-0.15	0.383	34	**-0.56**	**0.018***	17	0.26	0.311	17
Mono CI+II OXPHOS	Age (years)	0.04	0.805	37	0.23	0.334	20	-0.15	0.564	17
Mono CI+II OXPHOS	BMI (kg/m²)	**-0.40**	**0.013***	37	0.01	0.975	20	0.09	0.746	17
Mono CI+II OXPHOS	MVPA (min/day)	-0.23	0.199	34	-0.22	0.397	17	-0.34	0.176	17
Mono CI+II OXPHOS	Moderate PA (min/day)	**-0.31**	**0.075#**	34	-0.36	0.152	17	-0.14	0.595	17

Mono CI+II OXPHOS	Vigorous PA (min/day)	0.04	0.811	34	0.07	0.800	17	-0.04	0.872	17
Mono CI+II OXPHOS	Light PA (min/day)	0.07	0.692	34	0.20	0.450	17	0.00	0.993	17
Mono CI+II OXPHOS	Inactivity (min/day)	-0.14	0.415	34	**-0.54**	**0.026***	17	0.25	0.340	17
Mono ETS	Age (years)	0.04	0.814	37	**0.44**	**0.054#**	20	-0.34	0.175	17
Mono ETS	BMI (kg/m²)	-0.25	0.140	37	0.04	0.883	20	**0.51**	**0.036***	17
Mono ETS	MVPA (min/day)	-0.22	0.212	34	-0.30	0.237	17	-0.15	0.574	17
Mono ETS	Moderate PA (min/day)	-0.27	0.126	34	-0.38	0.129	17	-0.02	0.927	17
Mono ETS	Vigorous PA (min/day)	**0.34**	**0.045***	34	0.38	0.131	17	0.33	0.198	17
Mono ETS	Light PA (min/day)	0.07	0.699	34	0.36	0.153	17	-0.17	0.514	17
Mono ETS	Inactivity (min/day)	-0.02	0.914	34	**-0.51**	**0.036***	17	**0.43**	**0.082#**	17
**Muscle respiration × participant characteristics**										
Muscle Routine	Age (years)	**-0.30**	**0.088#**	33	**-0.60**	**0.009***	18	0.01	0.986	15
Muscle Routine	BMI (kg/m²)	0.02	0.895	33	-0.08	0.753	18	0.40	0.135	15
Muscle Routine	MVPA (min/day)	0.02	0.898	31	-0.18	0.499	16	0.27	0.339	15
Muscle Routine	Moderate PA (min/day)	-0.13	0.481	31	-0.32	0.228	16	0.04	0.873	15
Muscle Routine	Vigorous PA (min/day)	0.02	0.899	31	-0.32	0.221	16	**0.55**	**0.035***	15
Muscle Routine	Light PA (min/day)	**-0.39**	**0.031***	31	-0.28	0.302	16	**-0.51**	**0.050***	15
Muscle Routine	Inactivity (min/day)	0.16	0.379	31	0.15	0.568	16	0.17	0.532	15
Muscle Leak	Age (years)	**-0.38**	**0.028***	34	**-0.73**	**0.001***	18	-0.06	0.816	16
Muscle Leak	BMI (kg/m²)	0.12	0.486	34	-0.20	0.421	18	0.27	0.315	16
Muscle Leak	MVPA (min/day)	0.10	0.574	32	-0.17	0.527	16	0.35	0.177	16
Muscle Leak	Moderate PA (min/day)	-0.04	0.822	32	-0.31	0.248	16	0.11	0.674	16
Muscle Leak	Vigorous PA (min/day)	-0.04	0.813	32	**-0.52**	**0.039***	16	**0.61**	**0.012***	16
Muscle Leak	Light PA (min/day)	**-0.48**	**0.006***	32	**-0.43**	**0.096#**	16	**-0.57**	**0.022***	16
Muscle Leak	Inactivity (min/day)	0.25	0.162	32	**0.47**	**0.068#**	16	0.08	0.763	16
Muscle CI OXPHOS	Age (years)	-0.26	0.142	34	**-0.50**	**0.034***	18	-0.12	0.665	16
Muscle CI OXPHOS	BMI (kg/m²)	**0.42**	**0.012***	34	0.11	0.660	18	0.26	0.331	16
Muscle CI OXPHOS	MVPA (min/day)	0.02	0.927	32	-0.01	0.963	16	-0.07	0.791	16
Muscle CI OXPHOS	Moderate PA (min/day)	0.18	0.310	32	-0.08	0.756	16	0.21	0.441	16
Muscle CI OXPHOS	Vigorous PA (min/day)	-0.13	0.468	32	-0.32	0.222	16	0.08	0.772	16
Muscle CI OXPHOS	Light PA (min/day)	-0.04	0.831	32	-0.41	0.110	16	0.15	0.588	16
Muscle CI OXPHOS	Inactivity (min/day)	0.29	0.106	32	0.29	0.271	16	0.38	0.147	16

Muscle CI+II OXPHOS	Age (years)	**-0.35**	**0.044***	34	**-0.65**	**0.004***	18	-0.16	0.562	16
Muscle CI+II OXPHOS	BMI (kg/m²)	**0.36**	**0.039***	34	-0.11	0.671	18	0.30	0.260	16
Muscle CI+II OXPHOS	MVPA (min/day)	0.02	0.930	32	0.08	0.777	16	-0.15	0.586	16
Muscle CI+II OXPHOS	Moderate PA (min/day)	0.14	0.428	32	-0.06	0.829	16	0.11	0.685	16
Muscle CI+II OXPHOS	Vigorous PA (min/day)	-0.07	0.695	32	-0.28	0.294	16	0.17	0.529	16
Muscle CI+II OXPHOS	Light PA (min/day)	-0.11	0.558	32	-0.42	0.101	16	0.03	0.926	16
Muscle CI+II OXPHOS	Inactivity (min/day)	**0.31**	**0.088#**	32	0.29	0.281	16	0.41	0.117	16
Muscle ETS	Age (years)	-0.26	0.135	34	-0.38	0.119	18	-0.24	0.379	16
Muscle ETS	BMI (kg/m²)	**0.50**	**0.003***	34	0.31	0.212	18	0.22	0.405	16
Muscle ETS	MVPA (min/day)	0.02	0.914	32	0.10	0.713	16	-0.17	0.538	16
Muscle ETS	Moderate PA (min/day)	0.20	0.271	32	0.02	0.944	16	0.13	0.626	16
Muscle ETS	Vigorous PA (min/day)	-0.04	0.846	32	-0.17	0.541	16	0.12	0.659	16
Muscle ETS	Light PA (min/day)	0.01	0.944	32	-0.25	0.347	16	0.10	0.708	16
Muscle ETS	Inactivity (min/day)	0.22	0.218	32	0.11	0.696	16	0.39	0.132	16

Note: * p <0.05, # p<0.1

References

1. Tarnopolsky MA, Pearce E, Smith K, Lach B. Suction-modified Bergström muscle biopsy technique: experience with 13,500 procedures. Muscle Nerve. 2011 May;43(5):717–25. doi:10.1002/mus.21945 PubMed PMID: 21462204.

2. Janssen RC, Boyle KE. Microplate Assays for Spectrophotometric Measurement of Mitochondrial Enzyme Activity. Methods Mol Biol. 2019;1978:355–68. doi:10.1007/978-1-4939-9236-2_22 PubMed PMID: 31119674.

3. Fortes MAS, Marzuca-Nassr GN, Vitzel KF, da Justa Pinheiro CH, Newsholme P, Curi R. Housekeeping proteins: How useful are they in skeletal muscle diabetes studies and muscle hypertrophy models? Anal Biochem. 2016 Jul 1;504:38–40. doi:10.1016/j.ab.2016.03.023 PubMed PMID: 27060530.

4. Islam H, Tsai SH, Figueiredo C, Jackson GS, Marcotte-Chénard A, Bosak J, et al. Direct assessment of leukocyte signalling and cytokine secretion reveals exercise intensity-dependent reductions in anti-inflammatory cytokine action. J Physiol. 2024 Jun;602(12):2717–36. doi:10.1113/JP286228 PubMed PMID: 38776176.

5. Matthews DR, Hosker JP, Rudenski AS, Naylor BA, Treacher DF, Turner RC. Homeostasis model assessment: insulin resistance and beta-cell function from fasting plasma glucose and insulin concentrations in man. Diabetologia. 1985 Jul;28(7):412–9. doi:10.1007/BF00280883 PubMed PMID: 3899825.

6. Matsuda M, DeFronzo RA. Insulin sensitivity indices obtained from oral glucose tolerance testing: comparison with the euglycemic insulin clamp. Diabetes Care. 1999 Sep;22(9):1462–70. doi:10.2337/diacare.22.9.1462 PubMed PMID: 10480510.

7. Lyden K, Kozey SL, Staudenmeyer JW, Freedson PS. A comprehensive evaluation of commonly used accelerometer energy expenditure and MET prediction equations. Eur J Appl Physiol. 2011 Feb;111(2):187–201. doi:10.1007/s00421-010-1639-8

8. Troiano RP, Berrigan D, Dodd KW, Mâsse LC, Tilert T, McDowell M. Physical activity in the United States measured by accelerometer. Med Sci Sports Exerc. 2008 Jan;40(1):181–8. doi:10.1249/mss.0b013e31815a51b3 PubMed PMID: 18091006.
